# Bismuth-based nanostructured photocatalysts for the remediation of antibiotics and organic dyes

**DOI:** 10.3762/bjnano.14.26

**Published:** 2023-03-03

**Authors:** Akeem Adeyemi Oladipo, Faisal Suleiman Mustafa

**Affiliations:** 1 Polymeric Materials Research Laboratory, Chemistry Department, Faculty of Arts and Science, Eastern Mediterranean University, TR North Cyprus, Famagusta, via Mersin 10, Turkey

**Keywords:** advanced oxidation processes, emerging contaminants, low-dimensional nanomaterials, pharmaceutical by-products, Schottky junction

## Abstract

A serious threat to human health and the environment worldwide, in addition to the global energy crisis, is the increasing water pollution caused by micropollutants such as antibiotics and persistent organic dyes. Nanostructured semiconductors in advanced oxidation processes using photocatalysis have recently attracted a lot of interest as a promising green and sustainable wastewater treatment method for a cleaner environment. Due to their narrow bandgaps, distinctive layered structures, plasmonic, piezoelectric and ferroelectric properties, and desirable physicochemical features, bismuth-based nanostructure photocatalysts have emerged as one of the most prominent study topics compared to the commonly used semiconductors (TiO_2_ and ZnO). In this review, the most recent developments in the use of photocatalysts based on bismuth (e.g., BiFeO_3_, Bi_2_MoO_6_, BiVO_4_, Bi_2_WO_6_, Bi_2_S_3_) to remove dyes and antibiotics from wastewater are thoroughly covered. The creation of Z-schemes, Schottky junctions, and heterojunctions, as well as morphological modifications, doping, and other processes are highlighted regarding the fabrication of bismuth-based photocatalysts with improved photocatalytic capabilities. A discussion of general photocatalytic mechanisms is included, along with potential antibiotic and dye degradation pathways in wastewater. Finally, areas that require additional study and attention regarding the usage of photocatalysts based on bismuth for removing pharmaceuticals and textile dyes from wastewater, particularly for real-world applications, are addressed.

## Review

### Introduction

Worldwide, water pollution is rising, endangering the economic potential and development objectives of severely polluted areas because of the detrimental effects on human health and aquatic ecosystems. The improper disposal of industrial and agricultural pollutants (such as organic dyes, pesticides, and pharmaceutical residues) in water systems is becoming more and more of a global health threat. Over two billion people live in water-stressed countries, according to the World Health Organization (WHO, 2020), and it is anticipated that this situation will get worse in some areas because of the increased industrial discharge of contaminated water, population growth, and climate change [1]. According to current projections, 57% of the world's population will experience water shortages by 2050 if sustained and coordinated efforts are not made [2–3]. The estimate provided in [2–3] might have been too low. The projections for water consumption, availability, and quality are affected by a variety of unreliable geopolitical factors. Nevertheless, there is a growing need for the efficient removal of environmental pollutants and the proper treatment of industrial wastes to allowable discharge limits, which are crucial for preserving human life and protecting the environment.

Numerous techniques have been employed to treat contaminated water and wastewater, including adsorption, bioremediation, precipitation, electrocoagulation, filtration, membrane separation, flocculation, centrifugation, advanced oxidation processes based on photocatalysis, and chemical coagulation [4–11]. Each of these techniques has demonstrated varying levels of effectiveness and drawbacks that restrict their widespread use. For instance, due to deficiencies such as the formation of harmful by-products and incomplete removal of organic pollutants, traditional water treatment methods such as sedimentation, filtration, and precipitation, in particular, are believed to be ineffective [4,11]. As a result of the non-biodegradable and persistent nature of the majority of organic contaminants, some physicochemical treatment techniques, such as adsorption, are ineffective in removing them from water resources [11]. Because of their flexible design and low cost, biological approaches have been used for the treatment of various contaminated effluents. However, the process is time-consuming, can be ineffective when toxic recalcitrant pollutants are present, and may even be irreparably harmful to the environment.

Among the water treatment technologies, advanced oxidation processes (AOPs) are regarded as a practical, efficient, and fiercely competitive technology for water treatment for the removal of a variety of toxic and bio-recalcitrant organic pollutants and for the inactivation of pathogen microorganisms that cannot be treated by conventional methods [11–14]. For the oxidation of organic molecules, AOPs rely on the in situ generation of potent oxidants (reactive oxygen species, ROS) such as hydroxyl or sulfate radicals. AOPs have been broadly categorised in terms of how ROS are produced, including non-photochemical techniques, such as chemical, radiation-induced, cavitation, electrochemical techniques, and photochemical processes [11,15–17].

One of the AOPs, photocatalysis, uses natural light – a resource that is both clean and recyclable – to completely degrade a variety of organic pollutants and inactivate pathogens. The term “photocatalysis” refers to chemical reactions that use light and a photocatalyst (basically a semiconductor). A few of the requirements that an effective photocatalyst system should satisfy include high sunlight absorption, an appropriate gap (1.5–2.8 eV), long-term charge carrier separation, high photo-transporter mobility, appropriate physical and chemical properties, sufficient band alignment to meet the kinetic requirements of the target reaction, and anti-corrosion stability in reactive environments [18–20].

**Figure 1:**
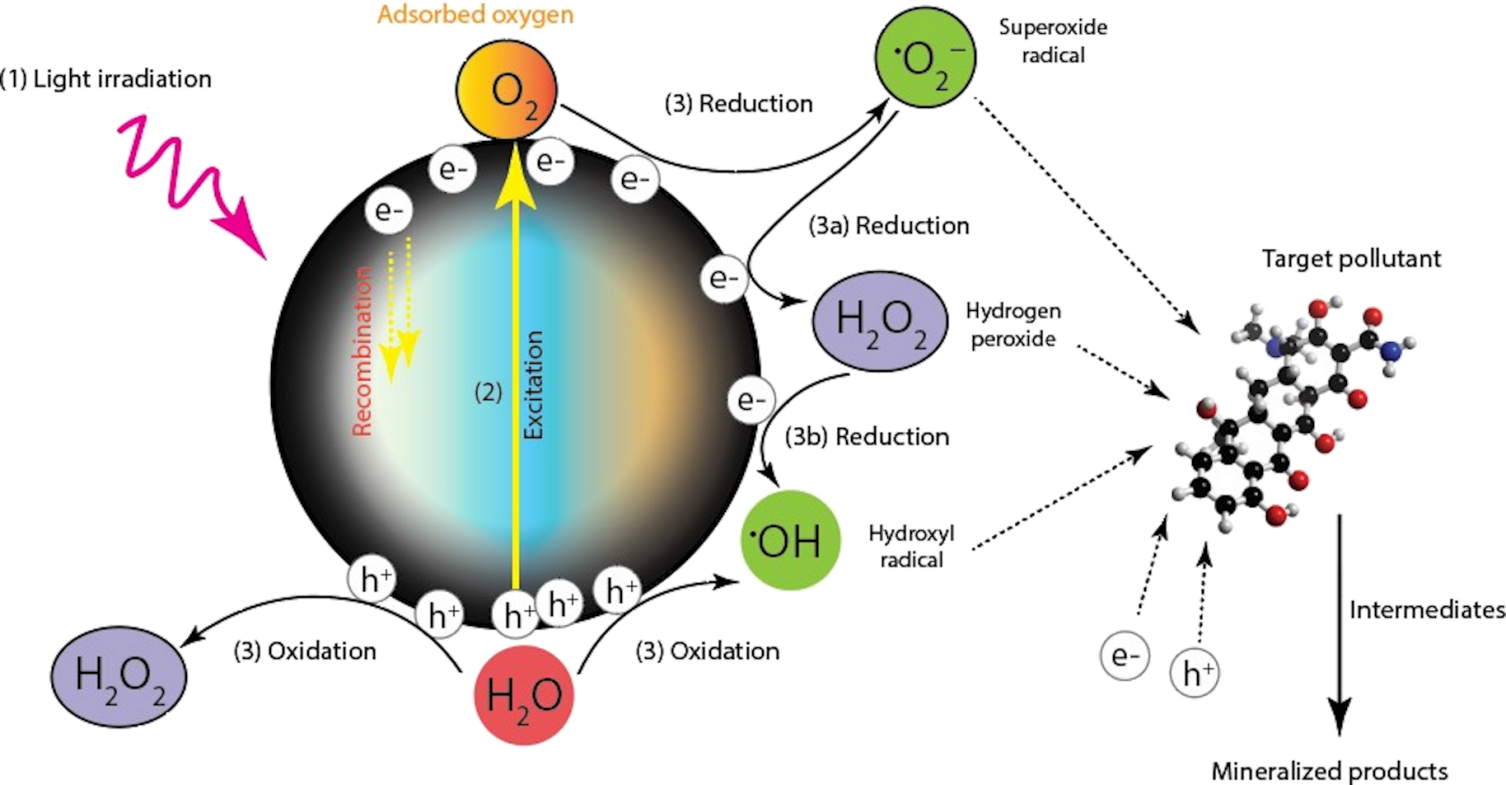
Mechanism of the photocatalytic process used to treat water contaminated with organic pollutants.

Figure 1 depicts the mechanism of the photocatalyst. In a nutshell, when exposed to light of the desired wavelength (enough energy), an electron (e^−^) in the photocatalyst's valence band absorbs photon energy and is excited to the conduction band on a femtosecond scale. This results in the formation of a hole (h^+^) in the valence band and a charge carrier pair (e^−^ and h^+^) on the surface of the photocatalyst. Three possibilities exist at this point: (a) The generated charge carriers recombine and generate heat, (b) the generated interfacial charge carriers simultaneously reduce and oxidise contaminants, or (c) the generated charge carrier and an electron donor or acceptor on the surface of the photocatalyst may continue to interact. Nothing happens in the first scenario. In the second scenario, an electron or hole interacts with dissolved oxygen or water to produce ROS (e.g., ^•^OH, O_2_^•−^). These ROS play a significant role in the photo-oxidation/reduction reaction, along with other species such as oxygen, hydrogen peroxide, and persulfate. This excited electron reduces an acceptor, and the acceptor's hole oxidises donor molecules. What happens to the excited electron and hole depends on the relative positions of conduction band and valence band of the semiconductor as well as the redox levels of the substrate [11,21].

One of the main barriers preventing photocatalysis from being used in practical applications is the lack of suitable semiconductor photocatalysts. The commonly used nanometre-sized photocatalysts are metal oxides or sulfides (binary compounds: TiO_2_, CuO, CdS, MoO_3_; ternary compounds: Bi_2_Mo_3_O_12_, ZnFe_2_O_4_; quaternary compounds: Ni_0.5_Zn_0.5_Fe_2_O_4_, Bi_4_Nb*_x_*Ta_1−_*_x_*O_8_I) [19–26]. Because of its distinct features, TiO_2_ is the most extensively investigated photocatalytic semiconductor. However, it barely absorbs 4–5% of the ultraviolet light in the solar spectrum due to its broad bandgap of 3.2 eV, which limits the use of visible light. Because of this, the potential photocatalytic use of TiO_2_ is constrained and the photocatalytic effectiveness is reduced [19–20,25]. Table 1 compares some of the salient characteristics of some of the bismuth-based photocatalysts with some of the typical metal oxide-based photocatalysts. Some of these important variables and values have been extracted from articles that have been published [27–38].

**Table 1:** Comparison of nanometre-sized metal oxide-based and bismuth-based photocatalysts.

Features	Metal oxides		

	TiO_2_	ZnO	SnO_2_

bandgap (eV)	3.0–3.4	3.10–3.37	3.76–4.24
performance based on the light source	very active in UV light	very active in UV light	very active in UV light
semiconductor type	n-type	n-type	n-type
crystal structure	anatase (tetragonal), brookite (orthorhombic), rutile (tetragonal)	hexagonal wurtzite (most stable at ambient conditions) and cubic zincblende	tetragonal
stability	photostable in solution and resistant to corrosion	readily dissolves in water, photocorrosion under UV	good stability
toxicity	nontoxic	low-toxicity	relatively non-toxic
photon absorption efficiency and quantum yield	high	higher than TiO_2_	moderate
cost	low	low	low
electron–hole pairs recombination rate	high	fast	high
magnetic properties	no	no	no

	Bismuth-based		

	BiFeO_3_	Bi_2_WO_6_	Bi_2_S_3_

bandgap (eV)	2.0–2.5	2.6–2.9	1.4–1.6
performance based on the light source	both visible and UV light	both visible and UV light	both visible and UV light
semiconductor type	n-type	n-type	n-type
crystal structure	rhombohedral distorted perovskite structure	orthorhombic	orthorhombic
stability	sufficiently stable	superior stability	highly stable
toxicity	low toxicity	nontoxicity	low toxicity
photon absorption efficiency and quantum yield	very high	moderately high	high
cost	low	low	low
electron–hole pairs recombination rate	high	fast	moderate
magnetic properties	ferromagnetic at low temperatures and superparamagnetic at room temperature. (multiferroic behaviour)	no	no

	Bismuth-based		

	BiOBr	Bi_2_O_3_	Bi_3_O_4_Cl

bandgap (eV)	2.69–2.99	1.5–2.8	2.6–2.8
performance based on the light source	both visible and UV light	both visible and UV light	both visible and UV light
semiconductor type	p-type	p-type	n-type
crystal structure	tetragonal (PbFCl-type structure)	monoclinic (room temperature), tetragonal β-phase or body-centred γ-phase (intermediate temperature), cubic (very high temperature)	cubic (Silleń structure)
stability	good chemical stability	highly chemically stable and photostable in solution	good stability
toxicity	nontoxic	low toxicity	nontoxic
photon absorption efficiency and quantum yield	moderately high	very high	moderate
cost	low	low	low
electron–hole pairs recombination rate	moderate	low	moderate
magnetic properties	no	paramagnetic behaviour	no

As an alternative to TiO_2_ for photocatalysis, nanometre-sized photocatalysts based on bismuth have recently been investigated and evaluated, because the majority of bismuth-based photocatalysts have a bandgap below 3.0 eV, making them usable in visible light. Additionally, their electrical structure produces a valence band with hybrid O 2p and Bi 6s orbitals, as opposed to the valence band of TiO_2_, which is made up entirely of O 2p orbitals. The mobility of the photogenerated charge carriers is increased by the well-dispersed Bi 6s orbital. Due to their distinctive structure, Bi-based photocatalysts exhibit a steeper absorption edge in the visible-light spectrum. Additionally, the reverse bond between the cation and anion is more favourable for the production and transportation of holes, which facilitates photocatalytic activity. Because of this, significant efforts have been made to synthesise bismuth-based nanomaterials (BiVO_4_, Bi_5_O_7_I-MoO_3_, Bi_2_O_3_, BiFeO_3_, Bi_2_WO_6_, Bi_2_Mo_3_O_12_, Bi_2_MoO_6_, and BiOI [24–25,39–45]) using a variety of techniques to tailor their size, morphology, and optoelectrical properties to improve their photocatalytic performance and to better understand the factors influencing their performance. Different materials based on bismuth have been developed and used for a range of environmental remediation applications. For instance, Mu et al. [46] synthesised a Bi_2_S_3_/Bi_4_O_7_ heterostructure via an in situ sulfidation approach and utilised it for the degradation of rhodamine B dye under visible-light exposure. Since the oxidation rate is still up to 96.3% after four cycles, the photocatalyst showed great performance and stability in the photocatalytic oxidation of the dye.

This review provides an overview of the recent nanostructured photocatalytic materials based on bismuth that are employed in the photocatalytic degradation of organic dyes and antibiotics in water. The general synthesis of nanometre-sized photocatalytic materials based on bismuth employing energy-efficient techniques is examined. A critical review is also given of ways to improve the photocatalytic activity of the photocatalysts. An extensive critical evaluation is given of recent findings on the photocatalysis of nanostructured materials based on bismuth and doped bismuth for the remediation of textile and pharmaceutical wastewater.

### Antibiotics and organic dyes in the environment and their toxicological consequences

Antibiotics are administered therapeutically to cure/prevent pathogen infections in people, animals, or both, as well as to increase livestock yields. However, since 50–80% of the antibiotic compounds that are taken are typically eliminated through urine and faeces, there are growing concerns regarding their excessive consumption and how they affect the environment. The widespread use of pharmaceuticals, especially antibiotics, has made them prevalent in the environment, and nearly everyone in the world now acknowledges their existence in both artificial and natural systems. Particularly, it has been claimed that antibiotic residues or metabolites have contaminated groundwater, soil, sediment, tap water, sludge, wastewater, and surface water.

Chemical manufacturing facilities, effluents from wastewater treatment facilities, and animal husbandry and aquaculture are the three main entry points for antibiotics into fresh waters [47–49]. According to the paper of Wise in the year 2002 [50], nearly 200,000 tons of antibiotics are consumed globally each year, with roughly 50% being utilized for veterinary medication and growth stimulants. Notably, between the years 2000 and 2010, the amount of antibiotics consumed by humans alone increased by 36% globally, demonstrating the ongoing problem of antibiotics pollution [51].

According to a recent study by Browne et al. [52], which covered 204 nations from 2000 to 2018, the rate of antibiotic consumption worldwide grew by 46% during the last 20 years. The report offers a comparative analysis of global human consumption rates of all antibiotics, expressed in defined daily doses (DDD) per 1000 population per day, a WHO metric. In contrast to the very low rates of consumption in sub-Saharan Africa and several regions of Southeast Asia, high rates of antibiotic usage were seen in the Middle East, Europe, and North America. The regions of South Asia (116% rise) and North Africa and the Middle East (111% rise) experienced the biggest increases in antibiotic usage rates. Specifically, in South Asia, third-generation cephalosporin consumption rates surged 37-fold and fluoroquinolone consumption rates increased 1.8-fold over the course of the study.

Different geographical areas have different levels of antibiotics in the environment. For instance, aus der Beek et al. [53] reported ofloxacin and sulfamethoxazole at 17.7 μg/L and 14.3 μg/L, respectively, and sulfamethazine has been reported with a concentration of 19 ng/L in Vietnam [54]. Sulfamethoxazole was lastly detected in Africa, where it was found at 53.8 ng/L in Mozambique [55] and 38.9 ng/L in Kenya [56]. Nalidixic acid and ciprofloxacin quantities of 23 μg/L and 14 μg/L, respectively, were found in South African streams and rivers [57]. While it is critical to understand the presence and levels of antibiotics in freshwater environments, it is maybe even more crucial to understand whether the residues or metabolites of the antibiotics have any impact on the various species that live there. The concentration necessary to produce a 50% effect after a given exposure time is known as the EC_50_. Chemicals having an EC_50_ between 10 and 100 mg/L are classified as hazardous, those from 1 to 10 mg/L as toxic, and those below 1 mg/L are classified as extremely toxic to aquatic life by the Commission of the European Communities [58]. The Wikipharma statistics [59] show that EC_50_ values were less than 1 mg/L in 25% of all research assessing the effects of antibiotics on eukaryotic, single-celled algae and that EC_50_ was even less than 100 μg/L in twelve investigations.

Once these antibiotics are released into the environment, non-target species are unavoidably exposed [47]. The development of antibiotic resistance, which has reduced the therapeutic capacity against human and animal infections, is the most significant issue associated with the release of antibiotics into the environment. It is not true that antibiotic resistance has never been observed in the natural environment; rather, it had previously only been linked to a small number of bacterial strains, but recent research has discovered antibiotic resistance genes in many other bacterial strains, raising serious health concerns. Antibiotic resistance is brought on by a high concentration of antibiotics that enter aquatic systems and interact with native species [47,60–62]. For instance, it may start to alter the genetic makeup and structure of the microbial community [47]. Antibiotic-resistant microbes (algae, fungi, and bacteria) pose a threat to both human and ecological health. The active ingredients of antibiotics and their fragments may cause kidney and liver cell damage in humans if they are exposed to antibiotic residues for an extended time [63–65]. Additionally, it has been noted that prolonged exposure to antibiotic-contaminated water might result in several allergic and respiratory conditions [62–65]. Additionally, an overabundance of antibiotics in the environment causes structural changes in the ecosystem, disruptions in ecological function, and impacts the processes of sulfate reduction, methanogenesis, and nitrogen conversion [61,63].

Antibiotics are persistent for long periods of time in natural environment. It is important to note that bacteria that develop resistance to one antibiotic also exhibit resistance to other drugs and chemicals. For example, Dickinson et al. [64] reported that the focal strain isolates from pond sediments in the northwest of the United Kingdom exhibited resistance to heavy metals and antibiotics (trimethoprim, oxacillin, and cefotaxime) where the *intI1* gene was involved. A growing body of research indicates that parent antibiotics and their metabolites, which are released into the environment in low concentrations (micrograms per litre to nanograms per litre), are persistent and bioactive, potentially posing a threat to the food chain.

Macrolides, fluoroquinolones, and tetracycline also have an impact on the synthesis of mitochondrial proteins and chloroplasts in plants [48,66]. Fluoroquinolones have a detrimental impact on the morphology and photosynthesis of plants, as well as on the ability of eukaryotic cells to synthesise DNA and replicate plastids. Streptomycin prevents *Hordeum vulgare* from producing chlorophyll, while ciprofloxacin, enrofloxacin, and sulfadimethoxine considerably slow down plant growth. Additionally, tetracyclines have phytotoxic effects that may result in chromosomal abnormalities and the reduction of plant growth. Although β-lactams are thought to be less harmful, they also have an impact on the plastid division in lower plants [48,67].

The textile industry, in addition to the pharmaceutical sector, is another sector that supports global economic expansion. It is one of the major sources of global pollution, although its importance cannot be disputed. Due to its high water demand when producing textiles and the limitations of conventional wastewater treatment techniques, the textile industry is causing concern. The direct release of textile waste into bodies of water without proper treatment to an acceptable level has a negative impact on its aesthetic quality. The presence of organic dyes in bodies of water, even in minute amounts, raises the chemical and biochemical oxygen demand and inhibits photosynthesis. Additionally, the uptake of dye molecules or their by-products in excess may be mutagenic, teratogenic, or carcinogenic [68–69]. Myocardial depression and hypertension are reportedly exacerbated by oral exposure to methylene blue dye. Additionally, some dyes, such as xanthene and erythrosine, have been related to allergic reactions, neurotoxins, and DNA damage in both humans and animals [70]. An eco-friendly, practical, and efficient treatment method is urgently needed because of the increasing pollution and health and ecological concerns of excess antibiotics and dyes in the environment. This article discusses the use of nanomaterials based on bismuth for the remediation of persistent organic pollutants.

### Bismuth and bismuth-based nanostructured photocatalysts

Bismuth (Bi) is a semimetal and a member of the p-block with a d^10^ configuration (6s^2^6p^3^) in the sixth period of group V of the periodic table. Because of their intriguing optical, catalytic, electrical, ferroelectric, and piezoelectric properties, bismuth-based nanostructures are used in several significant fields, including optoelectronics, pollutant sensing [71], and environmental remediation via photocatalysis [25]. Bi-based semiconductors, in particular, are thought to be able to surpass the limitation of the solar light-harvesting capacity of TiO_2_-based photocatalytic materials because of their smaller bandgaps. Because of its highly anisotropic Fermi surface charge, low carrier density, small electron effective mass, long electron mean free path, and extremely low band overlap energy, bismuth can transition from a semimetal to a semiconductor by shrinking its crystallite size [25,71–77].

**Figure 2:**
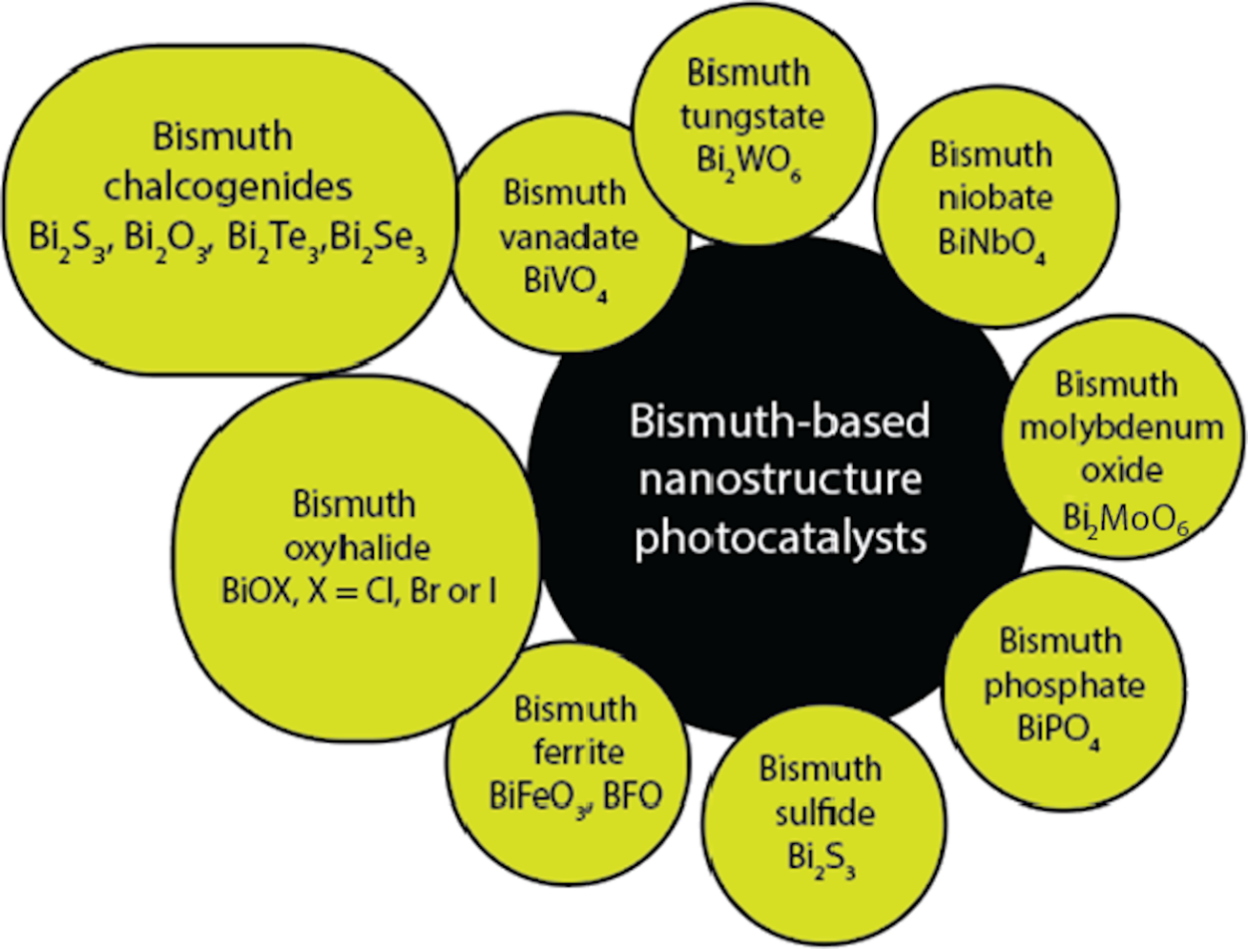
Most recently studied and common bismuth-based nanostructured photocatalysts.

To hasten the separation of photogenerated charges and, hence, increase photocatalytic activity, metallic bismuth can function as a direct plasmonic photocatalyst (similar to Au and Ag) or a co-catalyst [77]. Also, the unique layered crystal structure of Aurivillius-type bismuth oxide-based semiconductors allows for the induction of an internal static electric field, which effectively aids in the separation and transfer of photogenerated carriers. Bulk Bi and Bi-based nanostructure morphologies can also be easily altered using a variety of synthesis techniques due to their unique electrical and optical properties, which are directly tied to the plasmonic and photocatalytic properties. The typical and most recently applied bismuth-based nanostructure photocatalysts are depicted in Figure 2.

#### Structural, optoelectronic, and magnetic properties

Bismuth's peculiar optical, electronic, and more recently discovered photocatalytic and plasmonic properties have attracted the interest of a large community of scientists. With a low melting point of just above 544 K, Bi is less toxic than its neighbours in the periodic table, antimony, lead, and polonium. The structure of the bismuth crystal, which has rhombohedral symmetry, is typical of the group-V semimetals. Bi atoms form puckered bilayers of atoms perpendicular to the rhombohedral plane with three equidistant nearest neighbours and three equidistant next-nearest neighbours that are slightly farther away.

Bi is widely used in photocatalysis, in part because of its quantum confinement effect, which is important for electronic transport and semimetal-to-semiconductor transition, as well as its highly anisotropic Fermi surface (with an electron and hole Fermi energies of 27.2 and 10.8 meV, respectively), which results in an extremely low carrier density of around 3 × 10^17^ cm^−3^ [78] and very little overlap between the T-point band (valence) and the L-point band (conduction) [76–78]. Note that a reduction of the crystallite size below a critical value can result in a semimetal-to-semiconductor transition [77–80]. For instance, according to Qi et al. [81], indirect bandgap semiconductors were visible in Bi nanowires with a diameter of around 1–3 nm, but as the diameter increased, they became less visible because of the intense quantum confinement effect.

In addition to the electronic properties of Bi, its outstanding optical properties have a big impact on how effective it is as a photocatalyst. Bulk Bi exhibits high interband electronic transition rates that result in a negative ultraviolet–visible permittivity and a large infrared refractive index. Numerous investigations have shown that the quantum confinement effect affects the optical properties of Bi [25,71–80]. Furthermore, nanostructured materials exhibit unique optical properties that set them apart from the corresponding bulk materials as a result of this quantum confinement. Also, note that the optical responses of Bi nanoparticles are strongly influenced by their size, morphology, bandgap structure, shape, and environment. If these parameters are adjusted, the optical responses of Bi nanoparticles can be tuned from the near-ultraviolet to the near-infrared region.

**Figure 3:**
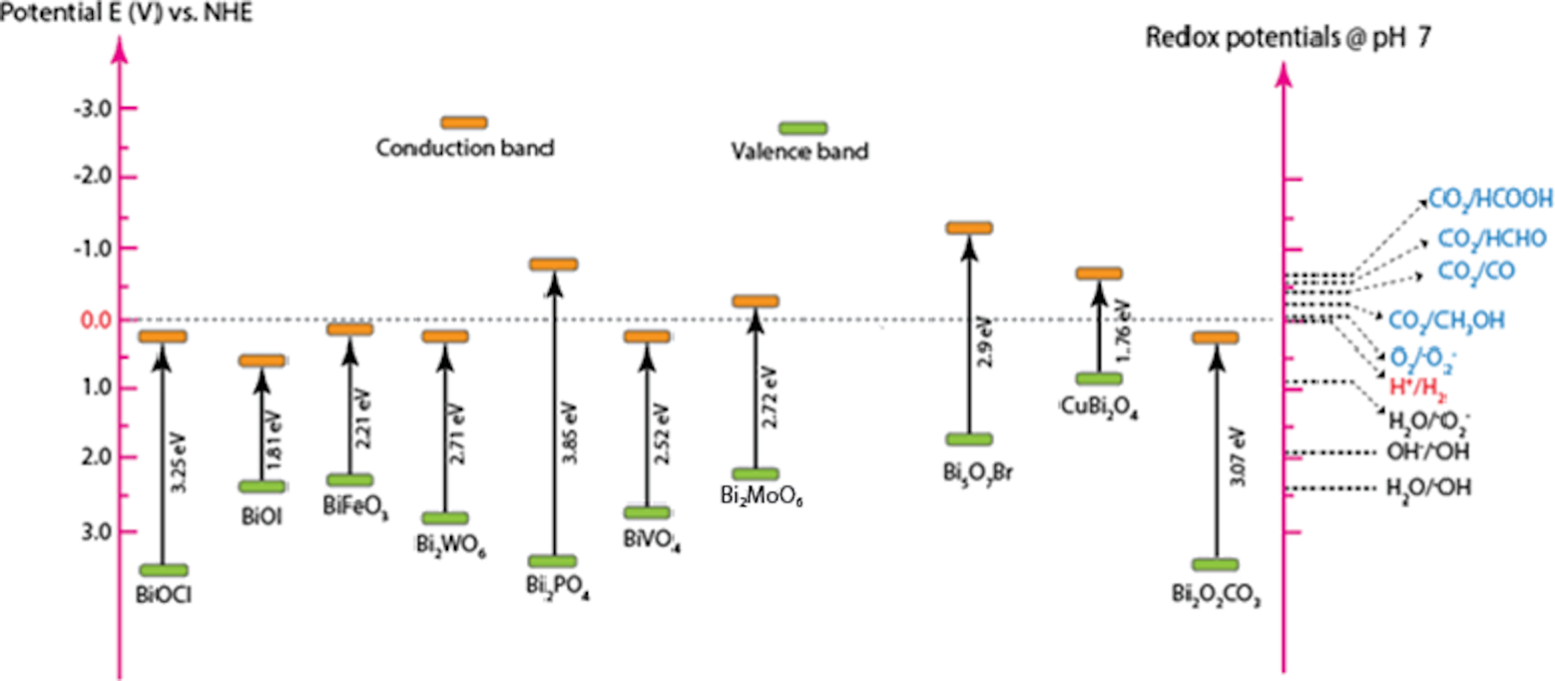
Bandgaps of some bismuth-based photocatalysts extracted from various research articles [27,35–37,83–86].

According to Figure 3, the bandgap of different bismuth-based photocatalysts has been observed to fall between 1.30 and 3.85 eV. From an optoelectronic structure standpoint, the majority of bismuth-based photocatalysts have a bandgap below 3.0 eV, which qualifies them for use in visible light. The hybridisation of the O 2p orbital and the 6s orbital in Bi is thought to be the cause of the narrow bandgap [82]. The valence band electrons are elevated by the hybridisation, which benefits the separation of photogenerated electron–hole pairs and the rate of charge carrier migration. Numerous visible-light photocatalysts based on bismuth have been used for the degradation of micropollutants because of their appropriate bandgap and non-toxic nature.

BiFeO_3_, one of those Bi-based photocatalysts, has been the subject of intensive research in recent years because it is the only naturally occurring magnetoelectric material with ferromagnetic and ferroelectric properties at room temperature [39,75,87–89]. Bismuth ferrite has a distorted rhombohedral perovskite structure (ABO_3_), where A is a corner cation, B is a body-centred middle atom, and O is an oxygen atom or anions attached to the crystal faces. BiFeO_3_ has strong magnetic and multiferroic, and sufficient photocatalytic properties due to this unique structure. BiFeO_3_ is an effective photocatalyst in the visible-light region, because in contrast to other semiconductors such as TiO_2_, it has a very narrow bandgap (Figure 3) and slow electron–hole recombination.

As 44% of solar radiation falls within the visible-light spectrum, BiFeO_3_ can be activated by direct sunlight, further lowering the cost of treatment. Aside from its magnetic and optical properties, BiFeO_3_ also exhibits piezoelectric characteristics, photovoltaic effects, switchable ferroelectric diode effects, and spontaneous polarisation enhancement. It is also sensitive to epitaxial strain [88]. Given its intriguing properties, a lot of researchers [90] have used bismuth ferrite to efficiently degrade organic pollutants, as shown in Table 2.

**Table 2:** Treatment of water containing antibiotics and dyes by bismuth ferrite nanoparticles (BiFeO_3_).

Particle size (nm)	Target pollutant	Source of light	Experimental conditions	Degradation (%)	Ref.
Remarks on the synthesis and main findings					

5.5	rhodamine B dye	visible light (high-power LEDs)	catalyst dosage: 1.25 g/L; solution pH 2; reaction time: 50 min; initial concentration of rhodamine B: 5 mg/L; observed bandgap: 2.07 eV	100.0	[75]
Monodisperse BiFeO_3_ nanoparticles were synthesised using a nanocasting approach, and they outperformed BiFeO_3_ nanoparticles prepared using other synthetic techniques in terms of photocatalytic efficiency and stability when exposed to visible light. When compared to particles of comparable size, the photocatalytic activity of the nanocast BiFeO_3_ particles is significantly higher. A low density of surface defects and few local strains contributed to this higher performance.					

35	rhodamine B dye	visible light (500 W Xe lamp)	catalyst dosage: 2 g/L; solution pH 0.5; reaction time: 60 min; initial concentration of rhodamine B: 10^−5^ mol/L; observed bandgap: 2.06 eV	100.0	[91]
By using a rapid sol–gel calcination approach, multiferroic BiFeO_3_ nanoparticles with rhombohedral crystal structures were synthesised, and they had stronger photocatalytic activity than the bulk. Mild room-temperature ferromagnetism was shown by the BiFeO_3_ nanoparticles.					

150–200	methyl orange dye	visible light (70 W 365 nm UV lamp)	with a catalyst loading of 6.4 mmol/L, the initial concentration of the methyl orange dye was 20 mg/L; the optimum reaction time was 260 min; the bandgap of the catalyst is 2.10 eV.	92.0	[92]
Chemical co-precipitation was used to synthesise the BiFeO_3_ nanoparticles, and analysis of the samples reveals that they have a perovskite structure that is distorted rhombohedrally and belongs to the polar *R*_3_*c* space group (no. 161). The nanoparticles' bandgap energy was lower than that of the bulk BiFeO_3_ (2.5 eV) due to the thinness of the sample.					

128	methylene blue dye	simulated solar light (Xe lamp 500 W)	catalyst concentration: 5 ppm; initial concentration of the dye: 1 ppm; pH 1–2; optimum reaction time: 50 min; the bandgap of the 1D nanofiber is 2.38 eV.	nanofiber 98.0 nanoparticulate 68.0	[93]
Electrospinning and the sol–gel method were used to synthesise BiFeO_3_ nanofibers and nanoparticles, respectively. According to the XRD findings, the BiFeO_3_ phase exhibits a rhombohedral structure with average crystallite sizes of 60 and 24 nm for BiFeO_3_ nanoparticles and nanofibers, respectively. Due to 1-dimensional confinements in the BiFeO_3_ nanofiber, its valence band edge position showed a shift toward higher energy, increasing its charge separation energy.					

5–50	congo red dye	visible light (Xe lamp, 500 W)	catalyst dosage: 2 g/L; initial concentration of the dye: 10 mg/L; optimum reaction time: 120 min; bandgap: 2.10–2.19 eV	95.0	[94]
The hydrothermal approach was used to synthesise the nanostructured BiFeO_3_ particles, which showed a single-phase perovskite structure. With a reduction in crystalline size, the band-gap energy of the nanoparticles increased.					

342–5560	tetracycline	visible light (500 W xenon lamp)	catalyst dosage: 0.5 g/L; initial concentration of the antibiotic: 10 mg/L; pH 8; optimum reaction time: 120 min; bandgap: 1.97 eV	77.0	[95]
According to the XRD data, the BiFeO_3_ particles showed a perovskite phase after being synthesised using a facile hydrothermal approach.					

20–150	oxytetracycline hydrochloride	visible light (300 W xenon lamp)	catalyst dosage: 1 g/L; initial concentration of the antibiotic: 20 mg/L; 10 g/L potassium peroxymonosulfate; pH 6.5; optimum reaction time: 10 min; bandgap: 1.78–1.95 eV	40.0–97.3	[96]
Through a mild one-pot hydrothermal procedure and a bath-ultrasound-aided dissolving technique, a multiferroic BiFeO_3_ photocatalyst was synthesised. The XRD analysis showed that the perovskite structure of the BiFeO_3_ nanocatalyst, which is composed of evenly spaced bimodal mesopores and nanoparticles smaller than 50 nm, was present.					

not given	cefixime trihydrate	direct sunlight	catalyst dosage: 20 mg/L; initial concentration of the antibiotic: 1 mg/L; pH 3 and 9; optimum reaction time: 30 min; bandgap: 1.72–2.25 eV	75.0–94.0	[97]
The combustion synthesis approach was used to synthesise the rhombohedral crystal structure in the bismuth ferrite nanoparticles. The typical crystallite size of the nanoparticles ranged from 24 to 48 nm, with various bandgaps.					

20	tetracycline	visible light (300 W mercury lamp)	catalyst dosage: 2 g/L; oxidant dosage (H_2_O_2_): 9.8 mmol/L; initial concentration of the antibiotic: 40 mg/L; pH 4; optimum reaction time: 210 min; bandgap: 2.1 eV	BiFeO_3_ alone 54.0 BiFeO_3_ + H_2_O_2_ 100.0	[98]
Sol–gel synthesis and calcination were used to synthesise the bismuth ferrite nanoparticles. The perovskite phase of bismuth ferrite was present in the nanoparticles, which were made up of almost rhombic nanoscale particles and showed no secondary contamination. The saturation magnetization value of 12.5 emu/g allowed the nanoparticles to be recovered and reused.					

17.4–929.6	doxorubicin	UV lamp	initial concentration of the antibiotic: 2 mg/L; optimum reaction time: 180 min; bandgap: 2.1 eV	79.0	[99]
Through the thermolysis of the coordination compound of bismuth ferrioxalate and calcination, bismuth ferrite particles were successfully produced. According to the XRD data, the bismuth ferrite nanopowders have a perovskite structure with rhombohedrally deformed (space group *R*_3_*c*) crystallites that range in size from 17.6 to 118.3 nm on average.					

9–16	ciprofloxacin and levofloxacin	simulated sunlight (500 W Hg Xe lamp)	catalyst dosage: 0.3 g/L; pH 3.5; initial concentration of the antibiotic: 10 mg/L; optimum reaction time: 240 min; bandgap: 1.18–1.95 eV	80.0 ciprofloxacin 79.0 levofloxacin	[100]
The BiFeO_3_ nanoparticles were synthesised at 160 °C by a simple high-pressure hydrothermal method and then doped with 10% gadolinium to facilitate the separation of electron or hole trapping sites and modify the band structures of the BiFeO_3_.					

Bi_2_WO_6_ is a typical Aurivillius-phase material, that is, a type of perovskite denoted by Bi_2_X*_n_*_–1_Y*_n_*O_3_*_n_*_+3_, where X is a large (12-coordinate, such as Ba, Bi, Sr, or Ca) cation and Y is a small (6-coordinate, such as Ti, W, Mo, or Fe) cation. It is another visible-light-driven n-type bismuth-based semiconductor and has received a lot of attention because of its distinctive layered structure, eco-friendliness, high photochemical and thermal stability, and benign visible-light photocatalytic activity [101–104]. Bi_2_WO_6_ has an orthorhombic structure, a high Curie temperature of about 900 °C, and a narrow bandgap of 2.6–2.8 eV [103]. Other desirable physical and chemical characteristics of Bi_2_WO_6_ include comparatively low toxicity, piezoelectricity, non-linear dielectric susceptibility, ferroelectricity, photostability and useful electrical properties [102–105]. The highest visible-light photocatalytic activity among bismuth-based oxides with a similar structure has been observed for Bi_2_WO_6_, which can be attributed to its distinctive structure [103–104].

It is important to note that several review articles [102–108] have covered in great detail different techniques used to synthesise Bi_2_WO_6_, its photocatalytic activities, strategies for altering its structure to increase photocatalytic performances, and its applications in environmental remediation. However, the goal of this review is to comprehend the most recent developments in the degradation of various textile dyes and antibiotics in wastewater using photocatalysts based on Bi_2_WO_6_. The use of Bi_2_WO_6_-based photocatalysts for the degradation of dyes and antibiotics has attracted great interest over the last eight years, as shown in Table 3.

**Table 3:** Bi_2_WO_6_-based nanostructured materials for remediation of antibiotics and dyes.

Morphology	Target pollutant	Source of light	Experimental conditions	Degradation (%)	Ref.
Remarks on the synthesis and main findings					

nanocrystals have uneven, rod-like, nanoplate, and nanoflower morphologies	ceftriaxone sodium	simulated sunlight (300 W Xe lamp)	catalyst dosage: 1 g/L; solution pH 2; optimum reaction time: 240 min; initial concentration antibiotic: 10 mg/mL; observed bandgap: 2.62 eV	70.18	[108]
By varying pH values (from 1 to 11), solvents (ethylene glycol and distilled water), and temperature (160–180 °C), different Bi_2_WO_6_ nanostructured materials were synthesised using a simple hydrothermal procedure. The findings showed that the morphologies effectively affected the photocatalytic activity of the samples. Due to its large surface area and improved light harvesting, the 3D flower-like structure made of ordered nanoplates provided the best antibiotic degradation efficiency.					

irregular nanocrystals with agglomerated nanocuboid morphology	levofloxacin	visible light (150 W Philips CFL bulb)	catalyst dosage: 0.75 g/L; solution pH 7.14; optimum reaction time:150 min; initial concentration of antibiotic: 10 mg/L; observed bandgap 2.61 eV	80.0	[109]
Bi_2_WO_6_ was synthesised using a hydrothermal process assisted by ultrasonication at 170 °C for 20 h yielding nanocuboids and orthorhombic phase crystal planes. The performance of the nanocuboids photocatalyst was enhanced by the presence of metal atom defects, crystal defects, or oxygen vacancies. The catalyst performs well at different pH values, although at natural pH, the maximum degradation was observed under visible light.					

flower-like microstructure morphologies with surfaces enriched with nanosized pores	norfloxacin and ciprofloxacin	visible light (300 W Xe bulb, CEL-HXF300)	initial concentration of antibiotic: 10 mg/L; catalyst dosage: 1 g/L; optimum reaction time: 150 min; observed bandgap: 2.36 eV	72.98–74.84	[110]
The Bi_2_WO_6_ photocatalyst was synthesised using the traditional hydrothermal process and autoclaved at 160 °C for 12 h. Superoxide radicals and photogenerated carriers (e^−^ and h^+^) are the main contributors to the degradation of antibiotics, while hydroxyl radicals have a very minor impact. Metal doping of the Bi_2_WO_6_ produced nanospherical structures with increased specific surface area, a narrower bandgap, and enhanced photocatalytic activity.					

flower-like, rod-like and lamellar-like nanostructures morphologies	enrofloxacin and norfloxacin	visible light (300 W Xenon arc lamp)	initial concentration of antibiotic: 10 mg/L; catalyst dosage: 0.5 g/L; solution pH 3; optimum reaction time: 75 min; observed bandgap: 2.57–2.85 eV	92.95–94.58	[111]
The Bi_2_WO_6_ nanorods were synthesised using a solvothermal process with ultrasonic assistance at 180 °C for 12 h, followed by 3 h of calcination at 350–550 °C. The calcination enhanced the crystallinity of the sample by producing nanocrystals with a greater ability to absorb visible light. The active species quench experiments revealed that h^+^ was the most significant active species in this study and that ^•^O_2_^−^ had a stronger degrading effect than ^•^OH.					

a hierarchical structure like a persimmon cake, with ultrathin nanoflakes of uniform size and morphology	norfloxacin	visible light (Xe bulb, CEL-HXF300)	initial concentration of antibiotic: 20 mg/L; catalyst dosage: 1 g/L; solution pH 9; optimum reaction time: 120 min; observed bandgap: 2.69–2.76 eV	95.0	[112]
A hydrothermal method was used to synthesise Bi_2_WO_6_ in a pH range of 4 to 11. Because of its higher specific area and rapid photogenerated carrier separation rate, ultrathin nanoflakes of Bi_2_WO_6_ produced at pH 4 demonstrated outstanding photodegradation effectiveness toward norfloxacin. The variations in the degradation rate were attributed to the different hierarchical structures of Bi_2_WO_6_.					

spherical shape aggregated perovskite nanoparticles	erichrome black T dye	simulated solar light (150 W Xe lamp)	initial concentration of antibiotic: 30 mg/L; catalyst dosage: 0.2 g/L; optimum reaction time: 180 min; observed bandgap: 2.7–2.9 eV	64.0–74.0	[113]
Using a one-pot solvothermal technique, Bi_2_WO_6_ nanoparticles were synthesised by changing the reaction temperature for 20 h between 120 and 180 °C. Because of the synergistic effects of small crystallite size, high surface area, presence of oxygen vacancies, and minimal electron recombination rate, the Bi_2_WO_6_ produced at 140 °C demonstrated maximum photocatalytic activity.					

sharp geometric corners and zigzag edges with nanoflakes morphology	Coomassie brilliant blue dye	visible light	initial concentration of antibiotic: 0.15 g/L; catalyst dosage: 0.75 g/L; optimum reaction time: 300 min	100.0	[114]
The hydrothermal method was used to synthesise orthorhombic Bi_2_WO_6_ nanoflakes for 24 h at 180 °C. The reaction temperature significantly affected the Bi_2_WO_6_'s morphology, as non-uniform morphology was seen at 180 °C. The well-defined nanoflakes were generated by continuing the reaction for longer periods of time.					

spherical, uniform, and well-developed 2D nanosheets with flower-like morphology	rhodamine B dye	—	initial concentration of antibiotic: 10 mg/L; catalyst dosage: 1.25 g/L; optimum reaction time: 40 min; ultrasound pulse mode: 9 s on/1 s off	99.5	[115]
The hierarchical Bi_2_WO_6_ nanostructures with a high surface area were synthesised using a hydrothermal technique at 180 °C for 2 h assisted by ultrasonication. Preparation temperature and time were crucial for the crystal development.					

disordered, flake-like nanocrystals	rhodamine B dye	UV light, visible light and simulated sunlight	initial concentration of antibiotic: 5 mg/L; catalyst dosage: 1 g/L; optimum reaction time: 180 min; bandgap: 2.97–3.0 eV	58.4–87.9	[116]
Bi_2_WO_6_ nanocrystals with an orthorhombic structure were synthesised by a hydrothermal method over a 24 h period at 200 °C. The as-prepared catalyst was loaded with NaBH_4_ solution (0.01–0.1 M) to enhance its structure and performance, and the optimized sample (0.03 M-Bi_2_WO_6_) showed the maximum photocatalytic activity. The Bi_2_WO_6_ nanoflakes have little photocatalytic activity when exposed to visible light because of their wide bandgap (3.0 eV), but are photocatalytically active when exposed to UV light. While exposed to UV and visible light, 0.03 M-Bi_2_WO_6_ (2.97 eV) exhibits increased photocatalytic activity. Due to its unique layered crystal structure of perovskite-like units (WO_4_)^2–^ positioned between (Bi_2_O_2_)^2+^ layers, Bi_2_WO_6_ nanoparticles exhibit strong photocatalytic performance.					

homogenous, bundle-like nanostructured morphology	methylene blue dye	visible light (tungsten lamp 250 W)	initial concentration of antibiotic: 5 ppm; catalyst dosage: 0.4 g/L; optimum reaction time: 180 min; solution pH 4; bandgap: 2.8–2.93 eV	79.1–87.7	[117]
By using a simple combustion process and jackfruit extract, Bi_2_WO_6_ nanoparticles were synthesised, which were subsequently calcined at 400 °C for 3 h. The synthesised nanocatalyst displayed an orthorhombic phase with a bundle-like structure and showed strong photoluminescence, photocatalytic, and antioxidant activity. Holes and hydroxyl radicals contributed significantly toward the degradation of the dye.					

Although Bi_2_WO_6_, BiFeO_3_, and other nanostructured photocatalysts [118–123] based on bismuth have been widely used in wastewater remediation and have demonstrated remarkable performance, their industrial/field application still faces some difficulties, including fast electron–hole pair recombination rate and challenges in separating them from the reaction system. Recently, a variety of methods have been used to enhance the photocatalytic activity. These methods include engineering their morphologies through various synthesis techniques, metal/non-metal doping, introducing heterojunctions, and combining them with other materials.

#### Synthesis approaches and performance enhancement

The majority of the reported photocatalysts have been used in laboratory settings. Several fundamental requirements must be met to produce an efficient photocatalyst that can be applied industrially for the remediation of a variety of pollutants in contaminated water. First, photocatalytic activity is significantly influenced by the morphology (e.g., nanoplates, nanotubes, nanowires, nanorods, nanocuboids, nanoflakes, nanosheets, nanocapsules, nanocasts, or nanodots), dimension, and particle size of the photocatalyst.

Functional properties such as bandgap, optoelectronic properties, surface area, photoresponse, and magnetic properties depend on particle size and morphology of Bi-based photocatalysts. For instance, the charge diffusion path can be decreased by using photocatalysts with extremely small or thin structures, effectively separating the photogenerated electrons and holes. Bi-based photocatalysts in zero to three dimensions have been developed recently [102,106].

Low-dimensional (0-D and 1-D) nanomaterials have been extensively used in the field of photocatalysis over the past few years due to their distinct optical and electronic characteristics [42,88,106]. Simple strain relaxation and short diffusion paths are benefits of 1-D nanostructured materials and are advantageous for the separation of photogenerated carriers [102]. 1-D spindle-like BiVO_4_ nanostructures with oriented carrier transport, high optical performance, and a short carrier diffusion length, for instance, were prepared by Li and co-workers [42]. The photodegradation rates of ciprofloxacin and tetracycline were, respectively, 94.8% and 81.1% after 1 h. Additionally, Lin et al. [122] prepared 1-D Bi_2_WO_6_ nanofibers with a flower-like morphology by using a hydrothermal process for the degradation of rhodamine B dye. Under visible-light irradiation, the 1-D nanofiber photocatalyst reached a degradation rate of 78.2% after 50 min.

Because of their extraordinarily small size, 0-D nanomaterials are steadily dispersed in solvents. There are very few published reports on 0-D Bi-based nanomaterials and these materials exhibit several quantum confinement effects. 3-D nanostructured Bi-based materials have drawn a lot of attention due to their intriguing architecture and properties. Numerous techniques have been developed to synthesise 3-D Bi-based nanostructures with different morphologies, including solvothermal/hydrothermal and sol–gel processes, mechanical exfoliation, solid-state reactions, chemical vapour deposition, and microwave-assisted techniques [106].

These 3-D photocatalysts have shown adequate photocatalytic activity, a large specific surface area, and an abundance of channels, all of which are advantageous for photocatalysis. For instance, Dang et al. [123] used a microwave-assisted method to synthesise 3-D nanostructured Bi_2_WO_6_ nanoparticles and reported 92% methylene blue dye degradation after 180 min under visible-light irradiation. 2-D nanostructured materials are thought to function more effectively in photocatalytic processes than 3-D nanostructured photocatalysts [88,102,106,124]. This is because photogenerated carriers in a 2-D structure can rise from a deeper layer of the structure more quickly than those in a 3-D structure.

**Figure 4:**
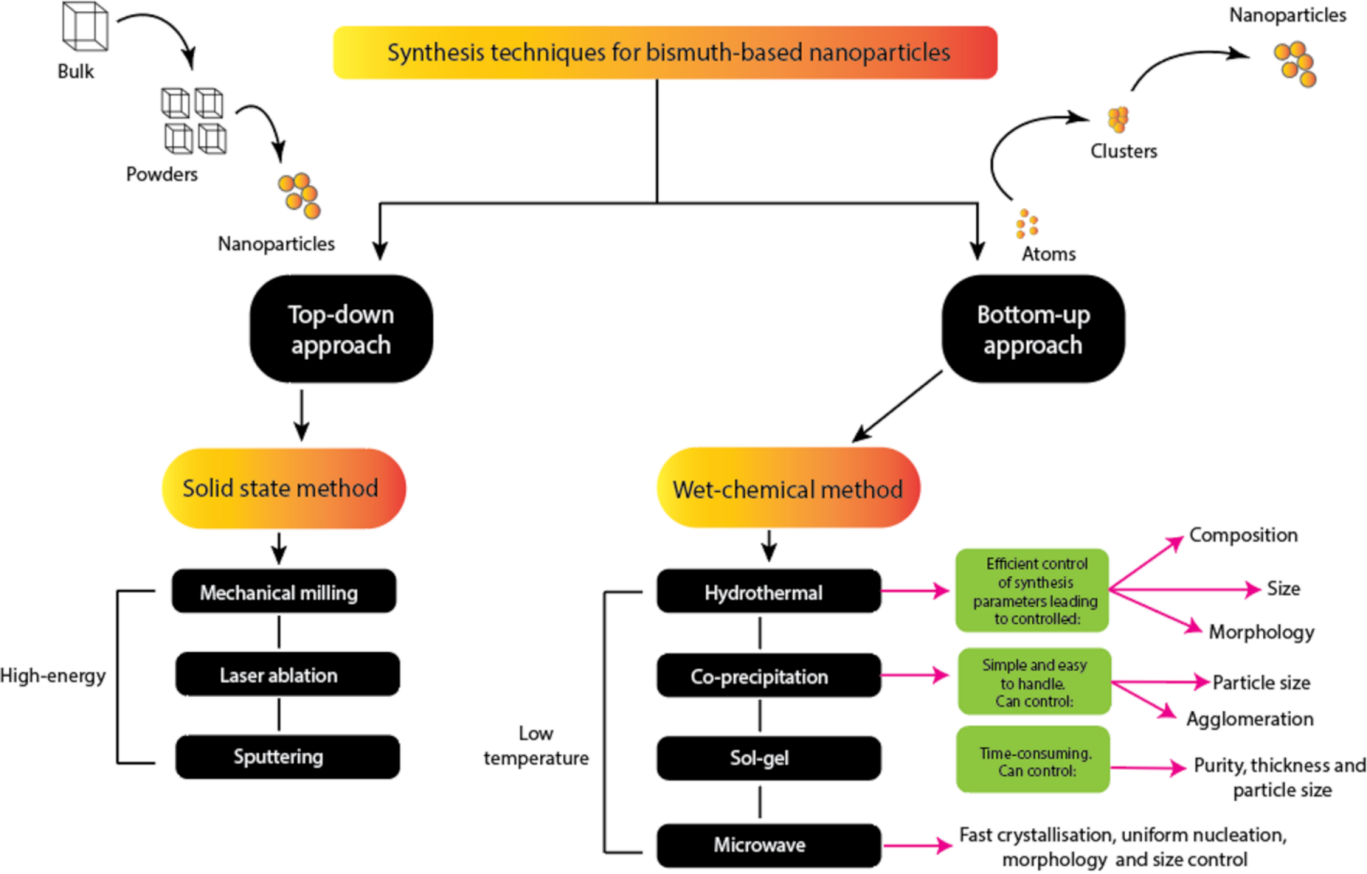
Summary of the commonly used synthesis methods for bismuth-based nanostructured photocatalysts.

It is important to note that an effective photocatalyst should have the following properties: (a) strong absorption both of UV and visible light (i.e., a suitable bandgap value, usually less than 3.0 eV); (b) thermal, chemical, and mechanical stability against photocorrosion; (c) high efficiency in quantum conversion; (d) fast generation and efficient transfer of photocarriers (e^−^ and h^+^); and (e) slow recombination rate of photogenerated charge carriers. The nanopowder photocatalysts must also exhibit easy and rapid recovery from the solution with adequate reusability, that is, without noticeable loss of efficiency. Several strategies are currently used to achieve the listed features, including tuning of size, morphology, and particle dimensions. Also, the composition of the photocatalyst is varied yielding core–shell structures, element substitutions, intercalation compounds, plasmon sensitization, heterojunctions, and composites [72,110,118–119]. Several synthesis techniques have been used as summarised in Figure 4.

Several synthesis procedures for bismuth-based photocatalysts have already been published [25,88,119–124], so they are not covered in this review. In general, top-down approaches or bottom-up approaches can be used to synthesise Bi-based nanostructured materials using traditional solid-state methods as well as wet-chemical methods. Solid-state methods are typically high-energy methods.

The final product might have some impurities, relatively large particles, and only a small degree of homogeneity. Large volumes of nanopowder can be produced using a relatively simple apparatus via solid-state routes. Wet-chemical methods (such as electrospinning, sol–gel, hydrothermal, ultrasound, co-precipitation, and aerosol-spraying) have been widely used for the synthesis of various nanostructured materials due to their low cost, low energy requirements, and ease of control of the solution parameters to meet the growing demand for efficient photocatalysts that can be produced on a large industrial scale at a lower cost. The most frequently used wet-chemical techniques are hydrothermal and co-precipitation techniques. Morphology, particle size, and composition can be easily adjusted using hydrothermal methods, whereas agglomeration and particle size can be controlled using the co-precipitation method. Thus, the combination of both techniques enables the customization of particle properties for particular applications.

It is worth noting that some recently published review articles have paid attention to ways of controlling the morphologies, dimensions, and even the nanoscale modulation as well as ways of enhancing the photocatalytic activities of Bi-based photocatalysts. Bi-based nanostructured materials have been used to treat water and wastewater that contained a variety of antibiotics (e.g., fluoroquinolones, tetracycline and sulfonamides). Table 4 and Table 5 discuss the results of a few studies that used undoped/non-composite Bi-based nanostructured photocatalysts to degrade textile dyes and antibiotics.

**Table 4:** Undoped Bi-based nanostructured photocatalysts for antibiotic remediation.

Photocatalyst	Target antibiotic	Optimum conditions	Source of light	Degradation (%)	Ref.
Remarks on active species					

BiOCl	ofloxacin, norfloxacin, ciprofloxacin	initial concentration of antibiotics: 10 mg/L; catalyst dosage: 0.25 g/L; optimum reaction time: 240 min	UV	95.0, 90.0, and 72.0	[125]
^•^OH radicals and h^+^ played key roles in the degradation process.					

BiVO_4_	ciprofloxacin	initial concentration of antibiotic: 10 mg/L; optimum reaction time: 120 min	visible light	58.6	[126]
While e^−^ and ^•^O_2_^−^ were the most active species, h^+^, ^•^OH and ^•^O_2_^−^ all took part in the degradation of the antibiotics.					

Bi_2_WO_6_	levofloxacin	catalyst dosage: 0.75 g/L; initial concentration of antibiotic: 10 mg/L; solution pH 7.14; optimum reaction time: 150 min	visible light	80.0	[109]
Antibiotics were degraded into simpler molecules as a result of the main active species, that is, radical ^•^O_2_^−^, h^+^, e^−^ and ^•^OH.					

Bi_2_WO_6_	norfloxacin, ciprofloxacin	optimum reaction time: 150 min; dosage of catalyst: 1 g/L; initial concentration of antibiotics: 10 mg/L	visible light	73, 70	[110]
^•^O_2_^−^ and holes (h^+^) played key roles in the degradation of the antibiotics.					

BiVO_4_	tetracycline	optimum reaction time: 60 min; dosage of catalyst: 1 g/L; initial concentration of antibiotic: 10 mg/L	visible light	monoclinic scheelite: 60.2 tetragonal zircon: 17.3 monoclinic-tetragonal: 80.5	[127]
Hydroxyl radicals and holes (h^+^) contributed to the degradation process					

BiOBr	ciprofloxacin	optimum reaction time: 60 min; dosage of catalyst: 0.5 g/L; initial concentration of antibiotic: 15 mg/L	visible light	43.7	[128]
While ^•^OH is only involved to a small extent in the antibiotic photodegradation, h^+^ and ^•^O_2_^−^ play a critical role.					

BiOCl	sulfamethoxazole	optimum reaction time: 60 min; dosage of catalyst: 0.2 g/L; initial concentration of antibiotic: 25 mg/L	visible light (Xe)	36.8	[129]
The main reactive species identified through scavenging tests were ^•^O_2_^−^ and ^•^OH.					

**Table 5:** Undoped Bi-based nanostructured photocatalysts for remediation of dye pollution.

Photocatalyst	Target dye pollutant	Optimum experimental conditions	Source of light	Degradation (%)	Ref.
Remarks on active species					

BiOI, Bi_2_O_4_	rhodamine B	treatment time: 32 min; dosage of catalyst: 0.5 g/L; initial concentration of dye: 10 mg/L	visible light (LED)	10.0, 67.0	[130]
^•^O_2_*^−^* and h^+^ were the main active species during the photocatalytic degradation process.					

Bi_4_Ti_3_O_12_	rhodamine B	treatment time: 120 min; dosage of catalyst: 0.1 g/L; initial concentration of dye: 10 mg/L	visible light (Xenon lamp)	100.0	[131]
^•^O_2_^−^ and h^+^ contributed mostly to the degradation process.					

BiVO_4_	alizarin red S	treatment time: 180 min; dosage of catalyst: 0.5 g/L; initial concentration of dye: 10 mg/L	UV (365 nm)	99.6	[132]
The degradation of the dye was mainly attributed to the contribution of ^•^OH radicals.					

BiOCl	rhodamine B	treatment time: 100 min; dosage of catalyst: 1 g/L; initial concentration of dye: 9.6 mg/L	visible light	22.0	[133]
The photogenerated electrons converted the adsorbed oxygen into ^•^O_2_^−^, which played a key role together with h^+^ in the degradation process.					

Bi_2_O_3_	acid yellow 29, Coomassie brilliant blue, Acid Green 25	treatment time: 120 min; dosage of catalyst: 1 g/L; initial concentration of Acid Yellow 29: 0.1425 × 10^−3^ mg/L, of Coomassie brilliant blue: 0.427 × 10^−4^ mg/L, and of Acid Green 25: 0.156 × 10^−3^ mg/L	visible light (Halogen lamp)	Acid Yellow 29: 58.0, Coomassie brilliant blue and Acid Green 25: 57.0	[134]
^•^O_2_^−^ and ^•^OH radicals were the key species while h^+^ contributed to the production of more ^•^OH radicals.					

Bi_2_O_2_CO_3_	rhodamine B	treatment time: 45 min; dosage of catalyst: 0.666 g/L; initial concentration of dye: 10 mg/L	visible light	13.0	[135]
^•^O_2_^−^ and ^•^OH radicals were the dominant species during the photocatalytic degradation process.					

BiOCl_0.7_I_0.3_	methyl orange	treatment time: 50 min; dosage of catalyst: 2 g/L; initial concentration of dye: 20 mg/L	visible light	100.0	[136]
—					

Bi_2_WO_6_	rhodamine B	treatment time: 100 min; dosage of catalyst: 1 g/L; initial concentration of dye: 4.8 mg/L	visible light (Xe light)	98.2	[137]
e^−^ and h^+^ contributed to the degradation process.					

BiOCl	methylene blue	treatment time: 120 min; dosage of catalyst: 1 g/L; initial concentration of dye: 20 mg/L	sunlight	36.0	[138]
^•^O_2_^−^ and ^•^OH radicals contributed to the photocatalytic process.					

BiOI	methyl orange	treatment time: 30 min; dosage of catalyst: 1 g/L; initial concentration of dye: 10 mg/L	visible light (300 W Xe lamp)	35.2	[139]
^•^O_2_^−^ and h^+^ were the dominant species while the ^•^OH radicals played a minor role in the degradation reaction.					

BiVO_4_	rhodamine B	treatment time: 180 min; initial concentration of dye: 10 mg/L	visible light (tungsten halogen lamp)	90.0	[140]
^•^O_2_^−^ and ^•^OH were the dominant species.					

BiOI	rhodamine B	treatment time: 240 min; dosage of catalyst: 0.25 g/L; initial concentration of dye: 10 mg/L	direct sunlight	100.0	[125]
The ^•^OH radicals and h^+^ played key roles in the degradation process.					

Bi_2_O_3_	methyl orange	treatment time: 240 min; initial concentration of dye: 10 mg/L	visible light	94.8	[141]
—					

BiFeO_3_	rhodamine B	treatment time: 180 min; dosage of catalyst: 0.2 g/L; initial concentration of dye: 10 mg/L	visible light	94.0	[142]
e^−^ converted O_2_ to ^•^O_2_^−^, which contributed actively to the degradation process alongside h^+^.					

Bi_2_WO_6_/AgIO_3_	rhodamine B	treatment time: 180 min; dosage of catalyst: 0.5 g/L; initial concentration of dye: 10 mg/L	visible light	100	[143]
The active species were ^•^O_2_^−^ and h^+^.					

Even though different Bi-based photocatalysts have demonstrated impressive photocatalytic performance, pristine and bulk Bi-based photocatalysts still have some drawbacks such as limited light absorption, weaker charge separation, and poor charge carrier mobility. Researchers are concentrating on several strategies, such as doping, heterojunction formation, induction of the surface plasmon resonance effect, and the formation of Z-schemes, Schottky junctions, and engineered composites, for modifying the optoelectronic and other properties of these Bi-based nanomaterials.

**Doping of Bi-based nanostructured materials:** To improve the electrical, optical, and magnetic properties of the host materials, doping (rare earth elements, metal, or non-metal ions) is a common technique [20,72,104,110,144–150]. Doping reduces the bandgap energy, introduces intermediate energy levels to overcome constraints, creates trap sites to capture photogenerated charge carriers, and increases the absorption of visible light. Additionally, after doping, oxygen vacancies or/and surface defects are created without destroying the crystal structure (though it might be distorted), effectively separating photogenerated carriers. Doping with metallic (Mg, Ag, Ni, Fe, Li, Co, and Ni) and non-metallic ions (F, C, N, and O), can introduce an intraband close to the conduction band of the host material, enhancing charge carrier dynamics [20,146].

In contrast to undoped Bi_2_WO_6_, visible light-driven 3-D hierarchical Ag-doped Bi_2_WO_6_ nanoparticles showed improved photocatalytic performance by destroying 95% of tetracycline in only 70 min, according to Shen and co-workers [147]. The increased performance was caused by the following factors: (a) surface plasmon resonance caused by the Ag dopant; (b) a decrease in the rate at which photoinduced carriers recombined; (c) high Schottky barriers between the Ag dopant and the host material; and (d) an increase in the visible-light absorption range. In addition to improving the photocatalytic properties of the Bi-based host materials, doping them with rare earth elements may also give them special ferroelectric and ferromagnetic properties, as well as electrochemical and luminescent properties.

To produce an Er-doped Bi_2_WO_6_ nanostructured photocatalyst for the degradation of antibiotics, Qiu et al. [145] used a hydrothermal technique. The bandgap of Bi_2_WO_6_ decreased from 2.80 to 2.35 eV after Er^3+^ doping, and the specific surface area of the doped Bi_2_WO_6_ was nearly 2.5 times higher than that of the undoped Bi_2_WO_6_. In comparison to pure Bi_2_WO_6_ (82.8%), the dopant significantly increased the tetracycline-degrading activity, which reached 94.6% within 1 h of visible light irradiation. Additionally, Irfan et al. [148] used a bi-solvent sol–gel technique to synthesise porous bismuth ferrite nanostructures with various morphological structures. They discovered that the surface area increased from 3.3 to 9 m^2^/g with a significant reduction in bandgap from 2.08 to 1.49 eV when La^3+^ and Mn^2+^ ions were co-doped into the BiFeO_3_ host material.

Within 120 min of exposure to visible light, the co-doped photocatalyst degraded Congo red dye by about 97%. The research on doped and co-doped Bi-based nanostructured materials using different dopants for dye and antibiotic degradation is summarised in Table 6 and Table 7.

**Table 6:** Doped Bi-based nanostructured photocatalysts for antibiotic remediation.

Photocatalyst	Target antibiotic	Optimum experimental conditions	Source of light	Degradation (%)	Ref.
Remarks on active species					

Mg-, Fe-, Cu-, and Zn-doped Bi_2_WO_6_^a^	norfloxacin, ciprofloxacin	treatment time: 150 min; dosage of catalyst: 1 g/L; initial concentration of antibiotic: 10 mg/L.	visible light	70.0–99.0	[110]
^•^O_2_^−^ and h^+^ were the dominant species while ^•^OH radicals contributed slightly to the degradation of the antibiotics.					

Al/BiOCl	tetracycline	treatment time: 60 min; dosage of catalyst: 0.4 g/L; initial concentration of antibiotic: 100 mg/L.	simulated sunlight	91.1	[149]
e^−^, ^•^OH and h^+^ played a minor role while ^•^O_2_^−^ was the main active species during the degradation process.					

Cu-doped BiOBr	norfloxacin	treatment time: 30 min; dosage of catalyst: 1 g/L; initial concentration of antibiotic: 10 ppm.	visible light (200 W Hg, Xe arc lamp)	46.5–82.6	[150]
The degradation of norfloxacin was mostly mediated by direct h^+^ oxidation; ^•^O_2_^−^ and ^•^OH radicals were not the predominant reactive species.					

Fe/BiOCl	levofloxacin	optimum reaction time: 60 min; dosage of catalyst: 0.5 g/L; initial concentration of antibiotic: 361 mg/L.	visible light	95.0	[151]
Both ^•^SO_4_^−^ and ^•^OH contributed little to the degradation of the antibiotic. ^•^O_2_^−^ and h^+^ were the main active species.					

Ti/BiOI	diclofenac	optimum reaction time: 90 min; dosage of catalyst: 0.25 g/L; initial concentration of drug: 10 mg/L; pH 5.9.	visible light	99.2	[152]
^•^O_2_^−^ and h^+^ were the key active species, while ^•^OH play a minor role during the degradation process.					

^a^The metals were doped individually.

**Table 7:** Doped Bi-based nanostructured photocatalysts for remediation of dye pollution.

Photocatalyst	Target dye pollutant	Optimum experimental conditions	Source of light	Degradation (%)	Ref.
Remarks on active species					

Ag-BiOCl	rhodamine B	treatment time: 100 min; dosage of catalyst: 1 g/L; initial concentration of dye: 9.6 mg/L	visible light	99	[133]
The photogenerated electrons converted adsorbed oxygen into ^•^O_2_^−^ radicals and with h^+^ contributed to the degradation process.					

Ce/Bi_2_O_3,_ Nd/Bi_2_O_3_	Acid Yellow 29, Coomassie brilliant blue (G250), Acid Green 25	Reaction time: 120 min; dosage of catalyst: 1 g/L; the initial concentration of Acid Yellow 29: 0.1425 × 10^−3^ mg/L., of Coomassie brilliant blue (G250): 0.427 × 10^−4^ mg/L, and of Acid Green 25: 0.156 × 10^−3^ mg/L	visible light (halogen lamp)	82.0–88.0, 74.0–84.0	[134]
^•^O_2_^−^, h^+^ and ^•^OH were the active species during the degradation process.					

B/BiOBr	rhodamine B	optimum reaction time: 30 min; dosage of catalyst: 1 g/L; initial concentration of dye: 15 mg/L	visible light	99.3	[153]
^•^OH played the main role in the degradation of rhodamine B.					

B/BiOCl	rhodamine B	optimum reaction time: 100 min; dosage of catalyst: 0.4 g/L; initial concentration of dye: 20 mg/L	visible light	81.5	[154]
^•^O_2_^−^ played the main role in the degradation of rhodamine B.					

C/BiOI	methyl orange	optimum reaction time: 60 min; dosage of catalyst: 0.1 g/L; initial concentration of dye: 10 mg/L.	visible light	99.8	[155]
^•^O_2_^−^ and holes played the main role in the degradation of methyl orange.					

In-BiOI	methyl orange	optimum reaction time: 120 min; dosage of catalyst: 0.1 g/L; initial concentration of dye: 10 mg/L.	visible light	96.0	[150]
^•^O_2_^−^ and holes played the main role in the degradation of methyl orange.					

The use of doping generally enhances the photocatalytic activity of photocatalysts. The most important variables to take into account are the quantity and type of dopant. The photocatalytic performance may be impacted if the amount of dopant is greater than the optimum value because it may act as a recombination site for photoinduced charge carriers. Additionally, doping has some drawbacks such as thermal instability and carrier trapping [72]. Other modifications, such as heterojunctions, Schottky junctions, p–n junctions, Z-schemes, and homojunctions, have been used to overcome these problems and boost the effectiveness of photocatalysts.

**Heterojunctions, Schottky junctions, Z-schemes and surface plasmon resonance effect:** Heterojunctions, which are the interfaces between two different semiconductors, increase the charge carrier separation efficiency with increased kinetics and strong redox ability. This enhances the photocatalytic capabilities of photocatalysts [101,119,156–161]. Depending on how the semiconductors are connected, heterojunctions can be divided into three types, namely type-I staggered gaps, type-II straddling gaps, and type-III broken gaps. In a broken gap, the bands do not overlap whereas in a staggered gap, the bandgaps of two semiconductors overlap and may cause band discontinuity. The straddling gap heterojunction system is recognised as the standard heterojunction system where the band edges of one semiconductor are lower than those of the second semiconductor [119,156]. The conduction band position of semiconductor Y is highly negative relative to semiconductor X in type-II heterojunction systems. Conversely, the valence band potential of semiconductor X is highly positive. After being exposed to visible light, electrons from semiconductor Y's conduction band move to semiconductor X's conduction band while holes from semiconductor X's valance band move to semiconductor Y's valance band, effectively separating the photogenerated carriers [162].

According to Chae et al. [163], a heterojunction WO_3_-BiVO_4_ composite demonstrated excellent photocatalytic activity with optical properties that were more effective than those of the pure individual components. Li et al. [164] also created a core–shell heterojunction nanocomposite made of BiFeO_3_ and TiO_2_ for the degradation of textile dye. The authors reported a 70% degradation of Congo red dye after 70 min of visible-light irradiation, which they attributed to an improvement in quantum efficiency caused by the efficient separating of holes and electrons. Charge carriers are oxidised and reduced at sites with reduced electric potential when they are separated by type-II heterojunctions, which, according to Low et al. [165], decreases the charge carrier separation efficiency and the redox ability of the photocatalyst.

The shortcomings of heterojunction systems have been overcome by Z-scheme photocatalysis systems, surface plasmon resonance effect, and Schottky junctions. An innovative method for further enhancing sunlight-driven photocatalytic performance in comparison to conventional heterojunction composites is to develop a Z-scheme photocatalytic system. Li et al. [42] constructed spindle-shaped BiVO_4_-RGO-*g*-C_3_N_4_ Z-scheme photocatalysts for the highly effective degradation of antibiotics. The 1-D Z-scheme ternary nanocomposites had a very high photooxidation response. According to the authors, ciprofloxacin and tetracycline were degraded by 94.8% and 81.1% after 1 h, respectively.

Another strategy for overcoming constraints such as low charge migration and the unpredictable direction of charge diffusion is the construction of a Schottky junction. A Schottky junction can be created at the interface between the semiconductor and a noble metal with an appropriate work function. A unidirectional charge transfer is enabled by the Schottky potential barrier, increasing charge density and separation [72]. Shen et al. [166] created a Schottky junction by synthesising NiSe_2_ nanosheets on top of BiVO_4_ nanosheets using a facile solvothermal technique. An intrinsic electric field is created at the interface as a result of the active migration of electrons from BiVO_4_ to NiSe_2_. This improves the separation efficiency of the photogenerated carriers, and the interaction at the interface lowers the bandgap of BiVO_4_, which in turn improves the photocatalytic activity of the nanocomposites.

Additionally, to maximise the effectiveness of the transfer/separation of photogenerated carriers, noble metals (such as Pt, Ag, and Au) are typically used to induce surface plasmon resonance effects in photocatalysts [146]. However, using noble metals in small or medium-sized industrial water treatment plants will be rather expensive. Recently, bismuth demonstrated a clear surface plasmon resonance effect, indicating the possibility of substituting it for noble metals. Because of the intrinsic photocatalytic characteristics of bismuth, other semiconductors can be used with it to achieve better performance. In a recent study, Chava et al. [167] synthesised bismuth quantum dots anchored to 1-D cadmium sulfide as a plasmonic photocatalyst using a facile solvothermal procedure. To create heterostructure nanorods, Schottky contacts between 1-D CdS and 0-D Bi components were developed. The bandgap values were altered, and the absorption in the visible-to-infrared range was enhanced after the deposition of Bi quantum dots on CdS. To degrade the antibiotic tetracycline, a Bi/CdS heterostructure photocatalyst was used. The optimised photocatalyst showed a maximum photocatalytic degradation activity of 90% under visible-light irradiation in 1 h, which is higher than the 52% achieved by pure CdS under the same conditions. The improved photocatalytic degradation efficiency is attributed to the surface plasmon resonance effect, doped Bi^3+^ ions, the Schottky potential barrier, and efficiently separated photoinduced charge carriers. Table 8 provides a summary of the research on heterojunction photocatalysts for the degradation of antibiotics.

**Table 8:** Bi-based nanocomposite/heterojunction photocatalysts for antibiotic remediation.

Photocatalyst	Target antibiotic	Optimum experimental conditions	Source of light	Degradation (%)	Ref.
Remarks on active species					

*Azadirachta indica* leaf extraction/BiOBr_0.2_I_0.8_	amoxicillin trihydrate	optimum reaction time: 300 min; dosage of catalyst: 1 g/L; initial concentration of antibiotic: 20 mg/L	visible light	93.2	[168]
The prime active species are h^+^ and ^•^O_2_^−^ while ^•^OH radicals play a minor role during the photocatalytic process.					

Bi_2_WO_6_/C-dots/TiO_2_	levofloxacin	optimum reaction time: 90 min; dosage of catalyst: 0.075 g/L; initial concentration of antibiotic: 10 mg/L	sunlight	99.0	[169]
The ^•^OH radicals play a key role in the degradation process while h^+^ and e^−^ contributed to the production of the active species.					

Ag/AgBr/BiVO_4_	ciprofloxacin	optimum reaction time: 120 min; initial concentration of antibiotic: 10 mg/L	visible light	91.4	[126]
Hydroxyl radicals, h^+^, and ^•^O_2_^−^ were the main species that contributed to the degradation process					

BiVO_4_/TiO_2_/RGO	tetracycline, chlortetracycline, oxytetracycline, doxycycline	optimum reaction time: 120 min; initial concentration of antibiotic: 10 mg/L, pH 3	visible light	96.2, 97.5, 98.7, 99.6	[170]
Both ^•^O_2_^−^ and ^•^OH were the key species that participated in the photocatalytic degradation process.					

*g*-C_3_N_4_/BiOBr on carbon fibre	tetracycline	optimum reaction time: 120 min; g-C_3_N_4_ nanosheets (thickness: ca. 30 nm, diameter: 0.4–1.0 μm) and BiOBr layer (thickness: ca. 25 nm, diameter: 200–500 nm); carbon fiber: (area: 5 × 5 cm^2^, weight: 0.15 g); initial concentration of antibiotic: 20 mg/L	visible light	86.1	[171]
^•^OH, h^+^ and ^•^O_2_^−^ were revealed to have participated in tetracycline degradation.					

Bi_2_O_3_–TiO_2_/activated carbon	sulfamerazine	optimum reaction time: 120 min; dosage of catalyst: 1 g/L; initial concentration of antibiotic: 20 mg/L	visible light	95.5	[172]
h^+^ and ^•^O_2_^−^ participated in sulfamerazine degradation.					

biochar@ZnFe_2_O_4_/BiOBr, biochar@BiOBr, ZnFe_2_O_4_/BiOBr	ciprofloxacin	optimum reaction time: 60 min; dosage of catalyst: 0.5 g/L; initial concentration of antibiotic: 15 mg/L	visible light	65.26, 47.1, 48.76	[128]
The results from the scavenger experiments revealed that radical h^+^, ^•^OH, and ^•^O_2_^−^ radicals contributed to the photocatalytic degradation process.					

AgI/Bi_4_V_2_O_11_	sulfamerazine	optimum reaction time: 60 min; dosage of catalyst: 1 g/L; initial concentration of antibiotic: 10 mg/L	visible light	91.47	[173]
^•^OH, h^+^ and ^•^O_2_^−^ were all involved in sulfamerazine degradation.					

BiOCl/*g*-C_3_N_4_/Cu_2_O/Fe_3_O_4_, BiOCl/g-C_3_N_4_/Cu_2_O, BiOCl/Cu_2_O/Fe_3_O_4_, BiOCl/g-C_3_N_4_/Fe_3_O_4_, BiOCl/g-C_3_N_4_, BiOCl/Cu_2_O	sulfamethoxazole	optimum reaction time: 120 min; dosage of catalyst: 0.2 g/L; initial concentration of antibiotic: 25 mg/L	visible light (Xe) and sunlight	Xe: 99.5; sunlight: 92.1, 85.3, 83.8, 80.7, 63.5, 59.4	[129]
The main reactive species identified through scavenging tests were ^•^O_2_^−^ and ^•^OH.					

Bi_2_WO_6_/g-C_3_N_4_	ceftriaxone sodium	optimum time: 120 min; dosage of catalyst: 1 g/L; initial concentration of antibiotic: 10 mg/L	visible light	94.5	[174]
h^+^ and ^•^O_2_^−^ radicals played a more significant role in the photocatalytic process then ^•^OH.					

AgI/BiOIO_3_	tetracycline, chlortetracycline	optimum reaction time: 350 min; dosage of catalyst: 0.5 g/L; initial concentration of antibiotic: 10 mg/L	visible light	tetracycline: 45.3, chlortetracycline: 39.1	[175]
From BiOIO_3_, h^+^ cannot sufficiently oxidise H_2_O molecules to form ^•^OH radicals. While the h^+^ in AgI oxidises OH^–^ to produce ^•^OH radicals, the electrons in AgI converted O_2_ to radical ^•^O_2_^−^. All contributed to the degradation.					

BiOBr/Bi_2_S_3_, BiOBr	ciprofloxacin, ofloxacin	optimum reaction time: 60 min; dosage of catalyst: 1 g/L; initial concentration of antibiotic: 20 mg/L	indoor fluorescent light	97.2, 89.28, 52.1, 44.21	[176]
^•^O_2_^−^ and h^+^ were shown to be the primary degrading species in scavenger experiments.					

**Bismuth nanocomposites:** A nanocomposite is a multiphase material (typically a solid) with one to three dimensions of less than 100 nm, where one of the phases has different properties due to differences in chemistry and structure. Following the formation of the nanocomposite, its properties are often enhanced and significantly different from those of the components. Fascinatingly, nanocomposite photocatalysts allow for the integration of multiple functions derived from various types of nanocatalysts, such as semiconductor nanoparticles, plasmonic metals, and carbon-based and magnetic oxides, into the same host matrix. This enables effective tuning of the photocatalytic characteristics of the final nanocomposite by extending the lifetime of the photogenerated carriers. It makes the catalysts recoverable by using external magnets and extends the range of absorption to the visible region for photocatalysis.

According to [177–180], a junction between carbon-based and semiconductor materials can effectively prevent charge carriers from recombining, increasing the photoactivity of BiFeO_3_. For instance, Wang et al. [180] synthesised spindle-like g-C_3_N_4_/BiFeO_3_ nanosheets, and the nanocomposite successfully degraded methyl orange by 75% under visible light, which is better than g-C_3_N_4_ or BiFeO_3_ alone. BiFeO_3_-graphene nanocomposites were made using a hydrothermal process by Lam and co-workers [177]. Under visible-light photocatalysis, the nanocomposite efficiently degraded Congo red dye. The improved performance was attributed to the altered bandgap between graphene oxide and BiFeO_3_. Table 9 provides a summary of the research on Bi-based nanocomposite photocatalysts for the degradation of dyes.

**Table 9:** Bi-based nanocomposite/heterojunction photocatalysts for remediation of dye pollution.

Photocatalyst	Target dye pollutant	Optimum experimental conditions	Source of light	Degradation (%)	Ref.
Remarks on active species					

BiOI/Bi_2_O_4_	rhodamine B	optimum reaction time: 32 min; dosage of catalyst: 0.5 g/L; initial concentration of dye: 10 mg/L	visible light	97.3	[130]
^•^O_2_*^−^* and h^+^ were the main active species during the photocatalytic degradation process.					

Bi_4_Ti_3_O_12_/C_3_N_4_	rhodamine B	optimum reaction time: 120 min; dosage of catalyst: 0.1 g/L; initial concentration of dye: 10 mg/L	visible light (xenon lamp)	100	[131]
^•^O_2_*^−^* and h^+^ were the main active species during the photocatalytic degradation process.					

Bi_2_O_2_CO_3_/g-C_3_N_4_	rhodamine B	optimum reaction time: 45 min; dosage of catalyst: 0.7 g/L; initial concentration of dye: 10 mg/L	visible light	91	[135]
h^+^ and ^•^OH were the main active species during the degradation process while ^•^O_2_*^−^* had only a small effect.					

*Azadirachta indica* leaf extract/BiOBr_0.2_I_0.8_	methyl orange	optimum reaction time: 80 min; dosage of catalyst: 1 g/L; initial concentration of dye: 20 mg/L	visible light	100	[168]
^•^O_2_*^−^* and h^+^ were the main active species during the photocatalytic degradation process.					

*Callistemon viminalis* extract/BiVO_4_	methylene blue	optimum reaction time: 300 min	visible light	82	[181]
^•^O_2_*^−^*, h^+^ and ^•^OH were the main active species during the photocatalytic degradation process.					

BiOCl_0.6_/ZnO_0.4_	rhodamine B	optimum reaction time: 140 min; dosage of catalyst: 1 g/L; initial concentration of dye: 40 mg/L	visible light (halogen lamp)	100	[182]
The main active species during the photocatalytic degradation process were ^•^O_2_^−^ and ^•^OH.					

BiOBr−BiOI	rhodamine B	optimum reaction time: 60 min; dosage of catalyst: 1 g/L; initial concentration of dye: 14.4 mg/L	visible light	90	[183]
The main active species during the photocatalytic degradation process were ^•^O_2_^−^ and ^•^OH.					

TiO_2_/Bi_2_O_3_	orange II	optimum reaction time: 180 min; dosage of catalyst: 1 g/L; initial concentration of dye: 5 mg/L	visible light (halogen tungsten lamp)	94.7	[184]
The main active species during the photocatalytic degradation process were ^•^O_2_^−^ and ^•^OH.					

BiOCl-Bi/TiO_2_	methylene blue	optimum reaction time: 120 min; dosage of catalyst: 1 g/L; initial concentration of dye: 20 mg/L	sunlight	97	[138]
Hydroxyl radicals, h^+^, and ^•^O_2_^−^ played key roles during the photocatalytic degradation process.					

BiOI/AgI/g-C_3_N_4_	methyl orange	optimum reaction time: 30 min; dosage of catalyst: 1 g/L; initial concentration of dye: 10 mg/L	visible light	95.2	[139]
Hydroxyl radicals, h^+^, and ^•^O_2_^−^ played key roles during the photocatalytic degradation process.					

Ag_8_W_4_O_16_/AgBiW_2_O_8_/Bi_2_WO_6_, AgBiW_2_O_8_/Bi_2_WO_6_	methylene blue	optimum reaction time: 40 min; dosage of catalyst: 1 g/L; initial concentration of dye: 10 mg/L	UV	82.5, 60	[185]
Considering the values of the potentials of the conduction and valance bands, e^–^, h^+^, and ^•^OH contributed to the degradation process.					

Au-BiVO_4_	methylene blue	optimum reaction time: 360 min; dosage of catalyst: 40 mL suspended nanoparticles/L; initial concentration of dye: 0.5 mg/L	UV–vis light	95	[186]
—					

Bi_2_WO_6_/g-C_3_N_4_	rhodamine B, methyl orange, methylene blue	optimum reaction time: 80 min; dosage of catalyst: 1 g/L; initial concentration of dye: 10 mg/L	visible light	99.9, 99.8, 99.8	[174]
h^+^ and ^•^O_2_^−^ contributed more to the photocatalytic process than ^•^OH.					

AgI/BiOIO_3_	methyl orange	optimum reaction time: 150 min; dosage of catalyst: 0.5 g/L; initial concentration of dye: 6.6 mg/L	UV and visible light	94.7, 48	[175]
Because the valence band of BiOIO_3_ has a lower potential than the redox potential of ^•^OH/H_2_O (2.38 eV), h^+^ cannot sufficiently oxidise H_2_O molecules to form ^•^OH radicals. While the h^+^ in AgI oxidises OH^–^ to produce ^•^OH radicals, the electrons in AgI can convert O_2_ to a radical ^•^O_2_^−^, which took part in the degradation.					

### Operational parameters influencing the photocatalytic efficiency of bismuth-based nanomaterials

In addition to the structure and properties of the photocatalysts used in pollution remediation, other crucial operational factors affect how well they perform. These parameters need to be investigated and optimised to scale up the process to design a system that is cost-effective, energy-efficient, and effective in treating water. Some of the factors that can affect the performance of the photocatalysts include the pH value of the effluent, the dosage of the photocatalyst, the initial concentration of the target pollutant, the dosage of oxidants, and the type of light source.

To lower the overall cost of water treatment, the photocatalyst must be effective under all types of light, including direct sunlight, UV light, and simulated sunlight. Several Bi-based photocatalysts are visible-light-driven because of the bandgap, making them useful in a variety of situations. The solution pH value is a critical parameter when it comes to the photocatalytic degradation of textile dyes and antibiotics. The point of zero charge (pH_pzc_) of the photocatalysts, the effluent matrices, and the speciation of the target pollutants at various pH values all affect how well the photocatalytic process degrades pollutants. To avoid additional cost associated with pH adjustment of the effluent, an effective photocatalyst needs to function excellently at all pH values. For instance, electrostatic repulsion may reduce the effectiveness of the degradation process if the photocatalytic experiment is carried out at a pH value at which photocatalyst and pollutant species have the same surface charge.

For instance, lower removal efficiencies for both ciprofloxacin and ofloxacin were recorded at a highly basic pH [187] using a magnetic Bi_2_WO_6_-biochar composite with a pH_pzc_ of 6.75. The best performance was at pH 7. Since both antibiotics and photocatalyst were negatively charged at a basic pH, electrostatic repulsion between them was thought to be the cause of this. A higher degradation efficiency was noted at basic pH using Bi_2_WO_6_ to degrade norfloxacin under simulated sunlight [188]. The higher removal was attributed to a potential reaction between the photogenerated holes and hydroxyl ions at basic pH, which may have produced more hydroxyl radicals, enhancing the photocatalytic reaction. These results unequivocally demonstrate the significance of the pH value in the degradation process and the necessity to fine-tune the photocatalysts to make them functional at all pH values.

The removal effectiveness and rate of photocatalytic degradation processes are significantly influenced by the amount of photocatalyst added to the effluent solution before treatment. In general, as the dosage of the photocatalyst is increased, the photocatalytic degradation efficiency rises as well, until an optimum point is reached where adding more photocatalysts has no further effect on the degradation efficiency. More active sites are provided in the effluent solution with the addition of more photocatalysts to the treatment reactor, which favours the production of more photoinduced carriers. The turbidity of the solution as well as the light scattering effect of the photocatalyst, however, may cause a decrease in the degradation efficiency when the amount of photocatalyst is above the optimal dose [157–162]. In addition to achieving maximum efficiency, using the optimum photocatalyst dose will also be very cost-effective.

Lower concentrations of antibiotics (nanograms per litre to micrograms per litre) have been found in environmental media, whereas the majority of textile effluents contain multiple pollutants at varying concentrations. Therefore, the photocatalyst and treatment system must be efficient to treat water with varying concentrations of contaminants. Both the effectiveness and kinetics of the photocatalytic processes are significantly influenced by the initial concentration of pollutants. Both direct and inverse relationships between the initial concentrations of the pollutants and the degree of their removal have been reported. Anwar et al. [189], for instance, reported that the photocatalytic degradation performance of both pollutants decreases with increasing initial concentrations of paracetamol and methylene blue dye. The decrease in the removal was attributed to the fact that high concentrations prevent light dispersion into the solution. An increase in the concentration of pollutant molecules adsorbed on the catalyst surface while the catalyst dosage is unchanged and the generation of reactive species is constant could be another factor causing the decrease in photocatalytic degradation rate with increasing concentrations.

A similar trend was reported by Huang and co-workers [190]. According to the authors, the percentage of photocatalytic degradation using a hierarchical Z-scheme AgBr-Bi_2_WO_6_ photocatalyst decreased from 88% to 54% when the concentration of tetracycline was increased from 20 to 60 mol/L. The decrease in efficiency was attributed to two causes: First, it is more difficult for photons to reach the photocatalyst at higher concentrations, which resulted in a decrease in the production of oxidant radicals and, as a result, a decrease in the degradation performance. Second, the number of intermediate products formed at higher concentrations increased, competing with tetracycline molecules and decreasing the efficiency of the photocatalytic reaction. To design a water treatment system that works effectively, the aforementioned parameters must be studied at both laboratory and industrial-scale reactors.

### Photocatalysis mechanism for bismuth-based photocatalyst and degradation pathway of target pollutant molecule

Understanding the degradation mechanism and the degradation pathway of the pollutants is crucial for designing an efficient photocatalyst and photocatalytic water treatment system. A less efficient and unstable photocatalyst may cause the nanocatalyst to leak into the environment and more hazardous intermediates to be produced. To understand the photocatalytic mechanism and speculate on potential heterojunction configurations for the photocatalysts, it is crucial to understand their optical characteristics, surface chemistry, and energy band structures. The structure of the molecule, the type and strength of its molecular bonds, the pH of the solution, the nature of reactive oxygen radicals, and the type of other pollutants in the system will all affect the degradation pathway.

Several simultaneous or sequential processes take place during the degradation of organic pollutants. The most frequently noticed reactions include the oxidative degradation of ring structures, hydration, dimerization, electron or charge transfer, hydroxylation, replacement, transformation, and rearrangement. In general, active reactive oxygen species (ROS) or photogenerated charge carriers may first remove a proton from a pollutant molecule or replace leaving groups such as halides for hydroxyl groups. Second, the bonds in organic pollutants that are particularly susceptible to degradation or those with less stereo-hindrance can be attacked by ROS or photogenerated charge carriers. Furthermore, smaller rings or cyclic structures, including three- to six-membered monocyclic compounds, are rapidly destroyed by ROS attacks due to the high ring strains. Radicals can combine to produce more resistant chemical species or unstable chemical species that can be further attacked by ROS to yield mineralized products.

**Figure 5:**
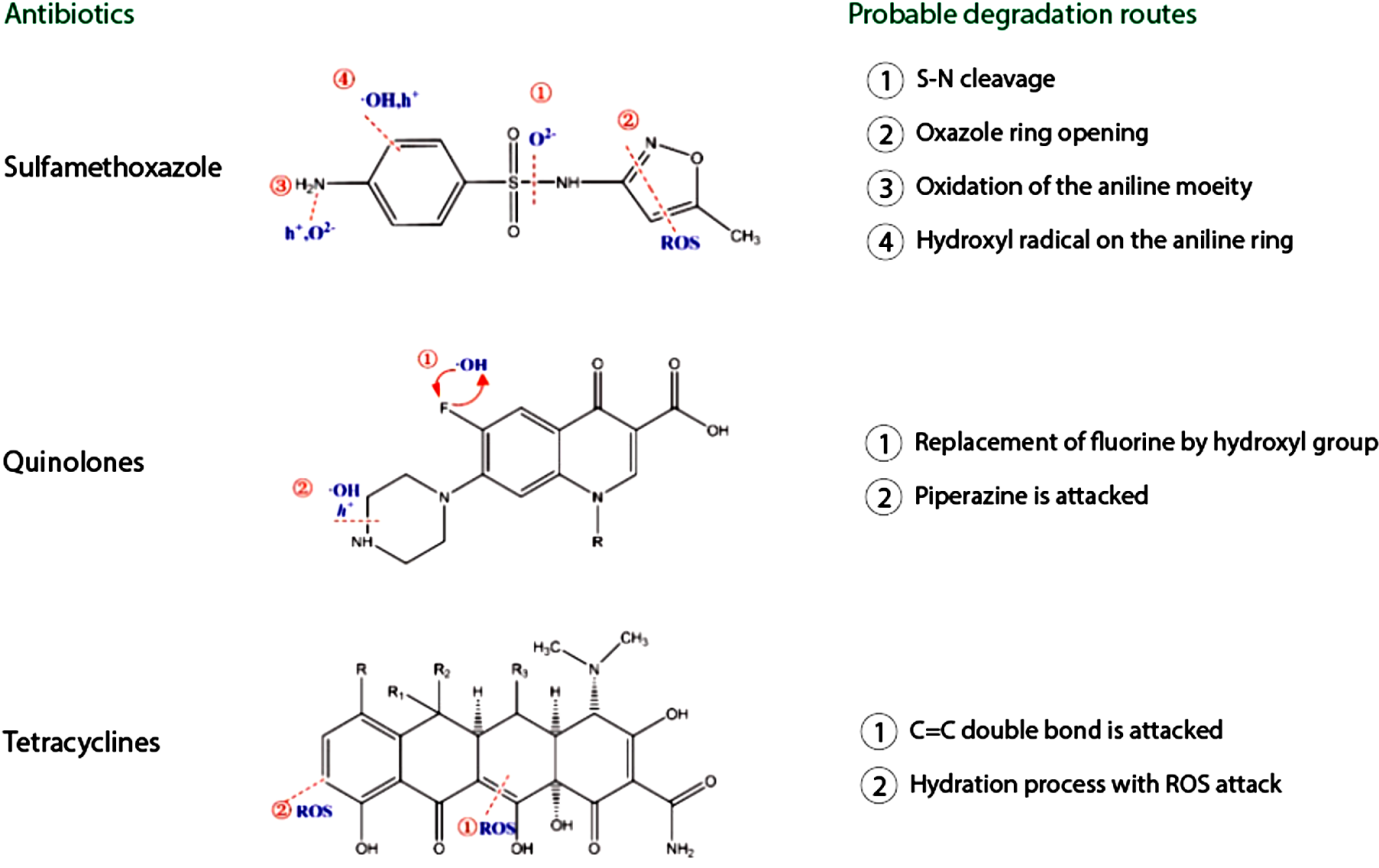
Photocatalytic degradation pathways of antibiotics by bismuth-based photocatalyst. (Adapted from [191], Environmental Research, Vol. 199, by K. Qin; Q. Zhao; H. Yu; X. Xia, J. Li; S. He; L. Wei; T. An, “A review of bismuth-based photocatalysts for antibiotic degradation: Insight into the photocatalytic degradation performance, pathways and relevant mechanisms “, Article No. 111360, Copyright (2021), with permission from Elsevier. This content is not subject to CC BY 4.0.)

For additional clarification, Figure 5 shows how typical antibiotics undergo bond breaking and degradation in bismuth-based photocatalysis. The antibiotic sulfamethoxazole, as an example, has a 4-aminobenzenesulfonamido group at the oxazole moiety's third position and a methyl substituent at the fifth position. The S–N bond is thought to be particularly susceptible to ^•^O_2_^−^ attack (indicated as route 1), and ROS attack can readily disintegrate the oxazole ring (marked as route 2). Meanwhile, numerous reports have also been made on the hydroxylation of the benzene ring and the associated NH_2_ (routes 3 and 4) [191–192].

Bi_2_WO_6_/AgIO_3_ nanosheets were synthesised using a two-step hydrothermal process, according to recent research by Liu et al. [143] for the photocatalytic degradation of rhodamine B dye. The researchers stated that the degradation of rhodamine B dye by Bi_2_WO_6_/AgIO_3_ nanosheets follows an S-scheme heterojunction mechanism (Figure 5a) during based on the electron spin resonance spectroscopy (ESR) result and relative energy band structure (valence and conduction bands).

**Figure 6:**
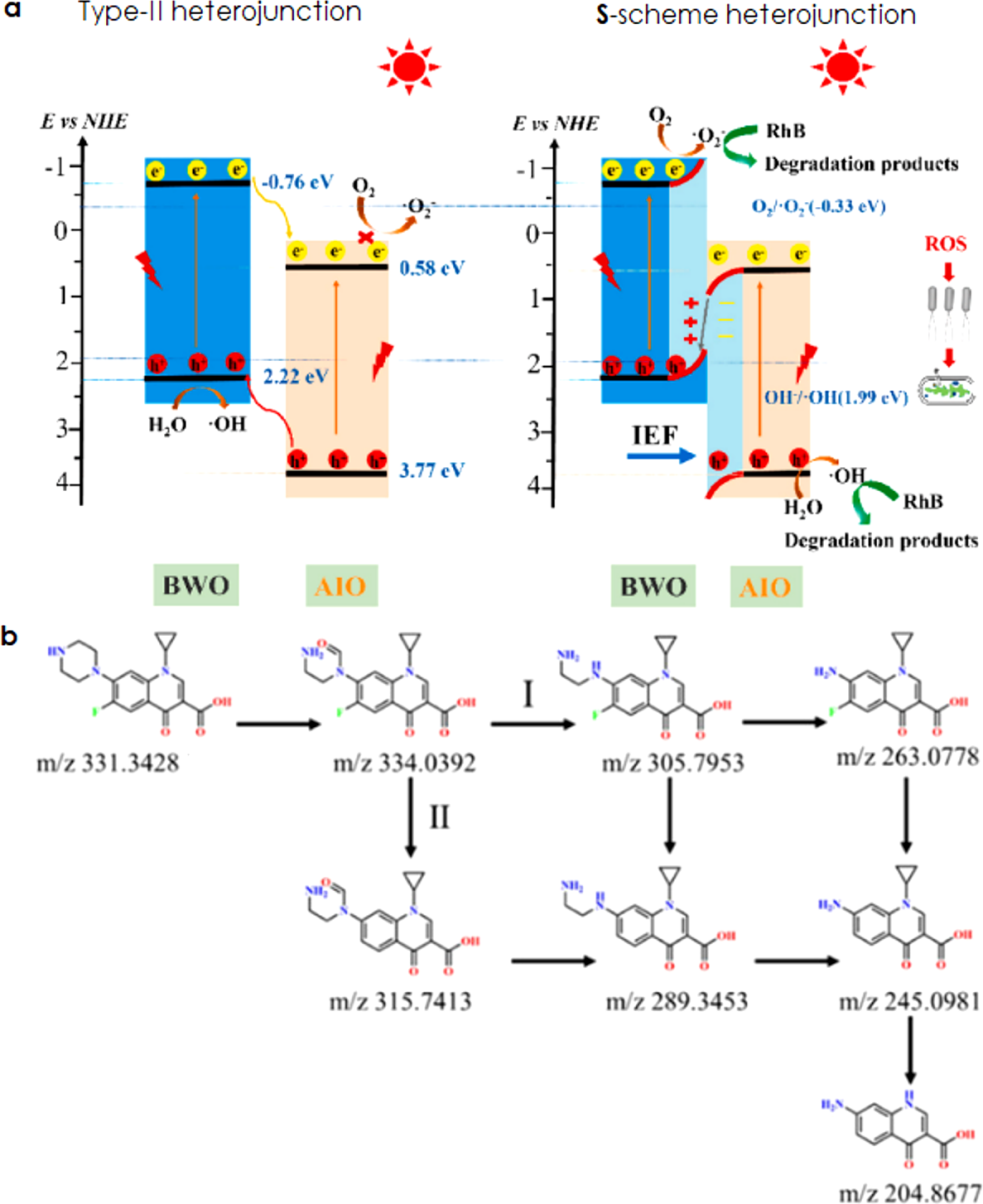
(a) Photocatalysis mechanisms of bismuth-based nanosheets via S-scheme heterojunction and type-II heterojunction systems (Figure 6a was adapted from [143], Ceramics International, Vol. 48, Issue 17, by Z. Liu; H. Wang; J. Duan; B. Hou, “An S-scheme heterojunction of Bi_2_WO_6_/AgIO_3_ nanocomposites that enhances photocatalytic degradation of Rhodamine B and antifouling properties“, Pages 24777–24787, Copyright (2022), with permission from Elsevier. This content is not subject to CC BY 4.0.). (b) The degradation pathways of ciprofloxacin by Mg-doped Bi_2_WO_6_ (Figure 6b was adapted from [110], Chemosphere, Vol. 252, by F. Zhu; Y. Lv; J. Li; J. Ding; X. Xia; L. Wei; J. Jiang; G. Zhang; Q. Zhao, “Enhanced visible light photocatalytic performance with metal-doped Bi_2_WO_6_ for typical fluoroquinolones degradation: Efficiencies, pathways and mechanisms“, Article No. 126577, Copyright (2020), with permission from Elsevier. This content is not subject to CC BY 4.0.)

The degradation mechanism may involve type-II heterojunctions, S-scheme heterojunctions, or Z-scheme heterojunctions, depending on the direction of electron–hole transmission. Specifically, when the Bi_2_WO_6_ and AgIO_3_ are activated by visible-light irradiation, electrons are transported from the valence band to the conduction band, leaving an equivalent number of holes in the valence band. The electrons in the conduction band (CB) of Bi_2_WO_6_ were moved to the CB of AgIO_3_ because its CB is more negative than that of AgIO_3_. In contrast, the position of the AgIO_3_ CB is greater than that of O_2_/^•^O_2_^–^ (−0.33 eV vs NHE). Based on this finding, AgIO_3_ CB electrons are unable to convert O_2_ to ^•^O_2_^–^, which is a limitation. However, the ESR results and radical trapping tests revealed that the major reactive radical in the photocatalytic experiments is ^•^O_2_^–^. Hence, Bi_2_WO_6_/AgIO_3_ is not compatible with the type-II heterojunction mechanism but with the S-scheme heterojunction mechanism. As seen in Figure 6a, when both semiconductors are in contact, the Bi_2_WO_6_ electrons migrate over the interface to AgIO_3_ until the Fermi energy levels are equal. At the interface, an intrinsic electric field is created to stop further electron transmission. As a result, the S-scheme heterojunction mechanism boosted the redox capability of the Bi_2_WO_6_/AgIO_3_ heterojunction, which greatly aided the photocatalytic decomposition of rhodamine B.

The degradation pathways for fluoroquinolone antibiotics (ciprofloxacin and norfloxacin) utilizing a metal-doped Bi_2_WO_6_ photocatalyst were identified by LC-MS/MS in another investigation by Zhu and co-workers [110]. In addition to additional peripheral moieties, ciprofloxacin has a quinolone moiety as its main functional group. The photocatalytic degradation of ciprofloxacin followed two pathways as depicted in Figure 6b and resulted in the identification of seven major intermediates by the authors. The loss of the –C_2_H_3_N group, the fluorine atom, and the formaldehyde group, as well as the oxidation of the cyclopropyl and piperazine groups, served as indicators of the intermediates.

The majority of studies on the degradation of dyes or antibiotics showed that these pollutants were effectively destroyed by the active species produced from compounds based on bismuth, but some researchers also insisted that those organic pollutants could not be fully mineralized and eventually converted into intermediates or metabolites. For instance, Chu et al. [193] found that despite a 97% removal rate being recorded, only 31% of the total organic carbon was eliminated after 6 h of irradiating tetracycline (20 mg/L) with 0.5 g/L of Bi_2_WO_6_. By using LC-MS/GC-MS, a total of eight intermediates were identified. The primary intermediates were thought to be the by-products of the reaction between photogenerated hydroxyl radicals and holes, which led to the loss of amino, hydroxy, and *N*-methyl groups, as well as to a rearrangement process. Even though several reports have shown the degradation pathways inferred after analysis of the degraded products, these researchers have not reported the toxicity of the by-products, particularly for those that are not fully mineralized. Some of these intermediates may be more toxic than the parent compound. To clarify the transformation and toxicity of the intermediates of bismuth-based photocatalysts, more research is required.

### Issues, challenges, and potential solutions

There are still some difficulties with several types of nanostructured bismuth-based photocatalysts despite their outstanding performance and widespread application in water remediation. In addition to discussing some current problems and challenges, this article also offers some potential solutions.

1. **Solubility and stability at low pH:** One of the difficulties with some bismuth salts is that they are unstable at low pH values and have low solubility constants, making them insoluble in aqueous solutions. Mineral acids, such as nitric acid or sulfuric acid, usually dissolve these compounds. The bismuth salt has been dissolved by some researchers using strong acids with concentrations as high as 5 M, which may further compromise its stability. The use of a combination of low concentrations of mineral acids and organic acids, such as citric acid or acetic acid, to dissolve the commonly used bismuth salts should be investigated in further studies.
2. **Nature of light source used for photocatalysis:** Several reports have used laboratory-scale UV–visible light lamps or bulbs or solar light simulators as sources of irradiation for the Bi-based photocatalysts during photocatalytic remediation of polluted water. Even though the majority of Bi-based photocatalysts have bandgaps that are suitable for direct sunlight irradiation, very few studies have been performed using this type of illumination. As a result, it is recommended that direct sunlight be used for photocatalysis rather than artificial solar light since it offers a more practical application and uses less energy. The majority of researchers also did not compute or present the actual light intensity that reached the effluent solution during treatment. This is encouraged to make it simple to scale up laboratory reactors to efficient field and industrial treatment units. Additionally, the majority of the studies lack actual photographs and detailed descriptions of the reactors that were used. This merits careful consideration because they offer a reliable basis for comparing photocatalysts.
3. **Multiple pollutants in lab settings and real wastewater:** The majority of studies only use one antibiotic or dye solution, which is far from the truth because wastewater (real effluents) frequently contains a mixture of dyes, dispersing agents, multiple pharmaceuticals, heavy metals, suspended solids, and surfactants. Therefore, the focus should be placed on researching the photocatalytic degradation of real wastewater and multipollutant solutions at the laboratory scale.
4. **Insufficient experimental details:** It has been noted that some researchers fail to provide a thorough account of their experimental procedures. Information such as the initial concentration of the target pollutant, photocatalyst dosage, solution pH, and reactor specifications, is often missing, which makes it difficult to compare their work fairly to that of other researchers.
5. **Instrumental analysis for trace concentration:** To estimate the degree of antibiotic degradation, the majority of researchers used UV–vis spectrophotometers. However, this instrument is less accurate when estimating trace levels of antibiotics, and there is also a significant chance that the antibiotics will oxidise to form more toxic intermediates that cannot be detected by UV–vis spectrophotometers. The percentage of total organic carbon removed from the analyte solution must be measured for fair comparison and an accurate assessment of the degree of degradation because this is an important indicator of how much the antibiotics are being mineralized. Future research should also examine the photocatalyst performance in both trace and concentrated dye solutions when using Bi-based photocatalysts to purify dye-polluted water.
6. **Recovery of powdered photocatalysts and toxicity:** Some of the nanopowder catalysts may escape and be discharged into the environment when nanostructured Bi-based photocatalysts are used to remediate pollutants. The production of reactive oxygen species and radicals might be hazardous to living organisms. There is presently no information on the toxicity of nanostructured Bi-based photocatalysts. Therefore, it is advised to produce magnetically recoverable nanostructured photocatalysts and do additional research on their toxicity.
7. **Recombination rate of photoinduced carriers:** Another problem is the recombination of holes and electrons, which lowers the photocatalytic performance. Tuning the energy bandgap, creating Schottky junctions or type-II heterojunction systems, using the Z-scheme, modifying the morphology, or using surface plasmon resonance are some of the methods used to overcome this particular problem. Future research should concentrate on combining these strategies to create a stable and remarkably exceptional photocatalyst. The majority of the 0-D quantum dot photocatalysts exhibit luminescence and other distinctive characteristics. Therefore, more research should focus on the development and use of low dimensional Bi-based photocatalysts.

## Conclusion and Perspectives

Numerous studies have demonstrated that the distinctive physicochemical and optical characteristics and the electronic band structures of bismuth-based nanostructure photocatalysts yield extraordinary photocatalytic activity under both visible and UV light. Numerous bismuth-based photocatalysts have also been extensively studied for their potential in detecting contaminants in the environment and addressing energy issues. There are still several challenges despite their outstanding photocatalytic performance. Although significant work has gone into improving the photocatalytic activity of bismuth-based photocatalysts, significant constraints regarding their application in the field of photocatalysis cannot be overlooked. In addition to describing problems and recent developments in photocatalysis, this article critically evaluates recently published research on nanostructured bismuth-based photocatalysts specifically for the remediation of water contaminated with textile dye and antibiotics. Researchers working on photocatalysts driven by visible light for efficient treatment of emerging trace contaminants may find the review work to be a useful resource.

Here, we have covered the fundamental workings of the photocatalytic process as well as the specifications for efficient photocatalysts. Outstanding visible-light activity, high stability, the capacity to efficiently separate and transfer photogenerated carriers with a low recombination rate, non-toxicity, adequate reusability, facile separation, and recovery after use are all requirements for a reliable and efficient photocatalyst. BiFeO_3_, Bi_2_WO_6_, and Bi_2_S_3_ are a few of the bismuth-based photocatalysts that have fascinating physicochemical characteristics and favourable bandgap values (1.5–2.8 eV), which enables them to be activated by visible light in contrast to TiO_2_ and ZnO semiconductors, which are often used and have wide bandgap values (>3.0 eV). Though some of the bismuth-based photocatalysts have intriguing characteristics, they nevertheless have a few drawbacks, such as rapid charge carrier recombination, delayed charge carrier migration, and low light absorption. Innovative low-energy synthesis techniques, morphological modulation, surface engineering, and bandgap tuning have been used by various groups to reduce these limitations. In this review, recent methods for creating extremely effective Bi-based photocatalysts are explored, including the creation of hybrid Schottky junctions and Z-scheme heterosystems. The review also looks at other operational parameters affecting the photocatalytic processes of Bi-based compounds used in water treatment. Although the majority of the experiments with Bi-based photocatalysts have used solar light simulators at the laboratory level, more thorough research into the use of direct sunlight and larger reactors with full specifications is advised to scale up its use and commercialization. This review is expected to pave the way for scientists to further develop Bi-based nanomaterials for the use in other processes, such as pollution sensing, photovoltaic systems, green energy harvesting and conversion, and catalytic systems other than photocatalytic processes.
